# Coherent amplification and inversion less lasing of surface plasmon polaritons in a negative index metamaterial with a resonant atomic medium

**DOI:** 10.1038/s41598-021-82909-7

**Published:** 2021-02-10

**Authors:** Saeid Asgarnezhad-Zorgabad

**Affiliations:** Departement of physics, Sharif University of Technology, Tehran, 11165-9161 Iran

**Keywords:** Nanoscience and technology, Optics and photonics, Physics

## Abstract

Surface plasmon polaritons (SPPs) lasing requires population inversion, it is inefficient and possesses poor spectral properties. We develop an inversion-less concept for a quantum plasmonic waveguide that exploits unidirectional superradiant SPP (SSPP) emission of radiation to produce intense coherent surface plasmon beams. Our scheme includes a resonantly driven cold atomic medium in a lossless dielectric situated above an ultra-low loss negative index metamaterial (NIMM) layer. We propose generating unidirectional superradiant radiation of the plasmonic field within an atomic medium and a NIMM layer interface and achieve amplified SPPs by introducing phase-match between the superradiant SPP wave and coupled laser fields. We also establish a parametric resonance between the weak modulated plasmonic field and the collective oscillations of the atomic ensemble, thereby suppressing decoherence of the stably amplified directional polaritonic mode. Our method incorporates the quantum gain of the atomic medium to obtain sufficient conditions for coherent amplification of superradiant SPP waves, and we explore this method to quantum dynamics of the atomic medium being coupled with the weak polaritonic waves. Our waveguide configuration acts as a surface plasmon laser and quantum plasmonic transistor and opens prospects for designing controllable nano-scale lasers for quantum and nano-photonic applications.

## Introduction

Surface-plasmon polariton lasers and amplifiers^1,2^, also known as microscopic/nanoscopic sources of light are important for providing and modulating linear and nonlinear interactions within subwavelength scales^3,4^. These nanophotonic elements are valuable in designing quantum- and nonlinear-photonic technologies such as an SPP frequency-comb generator^5^ phase rotors^6^ and quantum information processors^7,8^. Recent material technologies for fabricating nanoplasmonic configurations^9,10^ provide opportunities to exploit SPP lasing and amplifying^11–13^ in a wide range of applications such as in biology^14^ and quantum generator^15^. However, producing coherent lasing and stable amplification of SPPs are challenging due to the need for a giant phase mismatch for generating unidirectional SPP launching^16^, providing a population inversion in nano-scales^17^ and overcoming high Ohmic loss^2^.

Nanoscopic light sources or nanolasers are also important elements in wide range of applications form imaging to biology and chemistry^18,19^. However, lasing SPPs are inefficient^20^ and this amplification for plasmonic waves depends on the high laser powers^21^, the dense concentration of the gain medium^12^, and well-designed nano-scale materials^22^. Introducing a high-input field commensurate with a low concentration rate of the active gain limits the amplification efficiency. Moreover, including a dense dipolar gain produces amplified spontaneous emission that reduces the surface plasmon lasing operation^21^. Consequently, gain media properties and high-input field power induce noises to plasmonic systems that limit the efficiency of SPP lasers in the quantum regime. These limitations are challenging and prevent the realization of SPP lasing in an experiment^20^.

Previous investigations demonstrate that the quantitative and qualitative descriptions of the plasmonic nanolaser^22^ are possible for only quasi-static effects such as synchronizing plasmon oscillations with external injected field^23^ and intensity-dependent frequency shifts^24^. These proposals indicate that the surface plasmon lasing is obtained for a nanoscopic dipole resonance or relaxations of the gain media^20^. On the other hand, an investigation also reveals that quantum coherence can significantly enhance the surface plasmon amplification for a silver nano-particle that is coupled to the externally driven three-level gain medium^25^. The presented experimental and theoretical schemes for realizing surface plasmon lasers are based on the stimulated emission of radiation, which is hard to achieve within nanoscopic scales.

Placing atomic medium in the interface between a metallic-like and a transparent dielectric layers provides coherent control of excited SPP waves^26^ that is important due to its wide applications such as ultra-low loss nonlinear polaritonics^27^, entanglement creation^28^, polaritonic rogue waves and breather formation^29^, nonlinear frequency conversion^30^ and logic gates^31^. This hybrid plasmonic waveguide employs electromagnetically induced transparency windows of the atomic medium to control the linear and nonlinear properties of the excited SPP waves, and provides control over quantum properties of the propagated SPPs such as temporal or spatial coherence, quantum dephasing manipulation, and hence is applicable for both quantum^8^ and nonlinear nanophotonics^32^. These quantum and nonlinear properties of the atomic medium can be exploited to produce intense coherent SPP beam using a low-input power field or low concentration of the dense media.

Ameliorating the need to high input power and dense concentration of the gain medium as the main limitations and developing a lasing scheme with low-intensity laser fields and without the need to population inversion, thereby provides the opportunity to exploit these nanoscopic sources of light as a coherent amplifier, fast modulators, and efficient nanolasers. By proposing an atomic ensemble and exciting a superradiant emission of radiation, it is shown that a weak probe field amplifies without the need for population inversion^33^. Recently, a proposal indicates that directional superradiant surface-plasmon polaritons can be launched in the interface between a graphene layer and a heralded atomic scheme^16^; however, the weak plasmonic field amplification is not investigated within the presented graphene plasmonic scheme.

Consequently, fundamental questions that may appear are whether SPPs can also be amplified without population inversion, whether amplification needs a high-power field, whether this intense SPP field is coherent and uni-directional, and what would be the spectral properties of this coherent amplification? We give affirmative answers to these open questions by devising an ultra-low loss quantum plasmonic scheme, that exploits SSPP emission for amplification of the weak SPP field. The inversion-less lasing and coherent amplification of a weak SPP field, are two important and fundamental concepts in the field of plasmonics that have not been realized within a nanoscopic device. This work is methodologically novel as we introduce the quantum properties of the atomic medium to produce coherent amplification of SPP, and also conceptually novel due to introducing parametric amplification to produce nanolaser. We elucidate these concepts in details and explore this devise’s application to field-effect plasmonic transistors^34^ and nanoscale quantum generators^35^.

**Figure 1:**
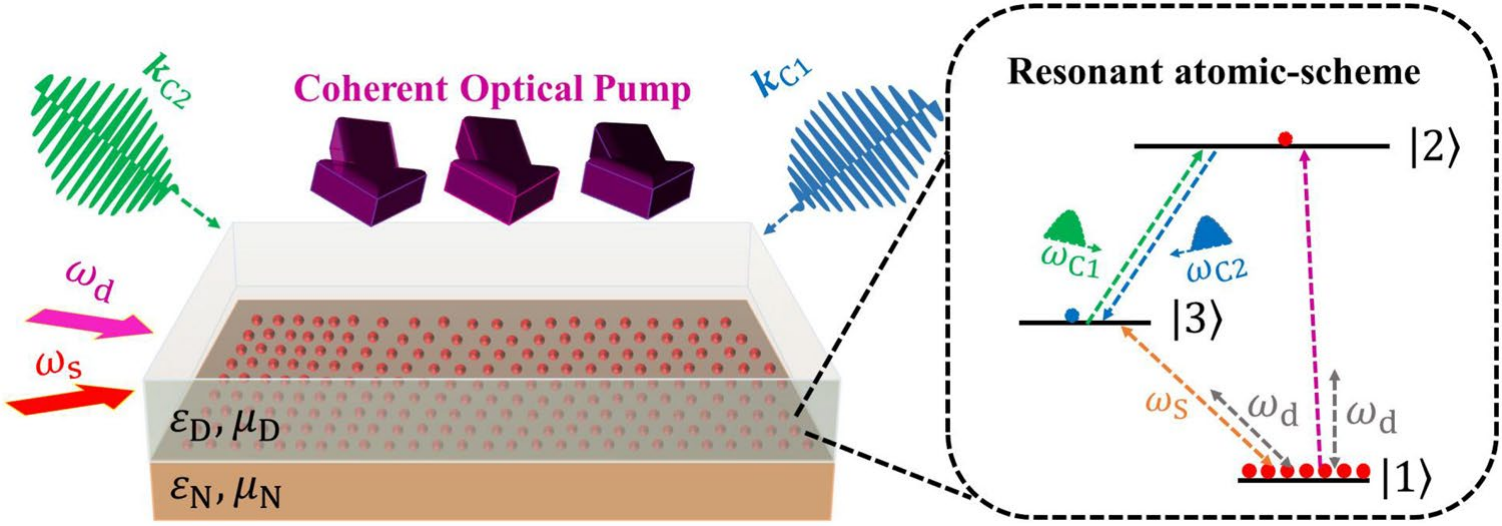
Waveguide configuration for the amplification of SSPP emission. The waveguide comprises a dielectric layer with electric permittivity $\varepsilon _\text {D}$ and a (NIMM) layer with optical constants $(\varepsilon _\text {N},\mu _\text {N})$. The atomic medium is doped in the dielectric-NIMM layer interface. A coherent optical pump and a train couple laser fields provide phase matching, that yield SSPP excitation. The inset of this figure represents the energy diagram of our proposed atomic medium. $| {l} \rangle$; $l\in \{1,2,3\}$ is the energy levels, $\omega _\text {d}$ is the central frequency of the driving field, and $\omega _\text {s}$ is the central frequency of the weak signal field. The coupled field enters to the system as a train of $\pi$-pulse with a central frequency $\omega _{\text {C}i}$ and wavenumber $\varvec{k}_{\text {C}i}$ for $i\in \{1,2\}$.

Now, we further elucidate the technical and conceptual dissimilarities between this work and other previous investigations. Technically, spasers and plasmonic nanolasers in other systems need population inversion that can be achieved with high input filed power^20,21^ and dense concentration of gain media^12,19^, which destroys spectral properties due to amplified spontaneous emission and unwanted inhomogeneous broadening. Here, we introduce an inversionless framework with modified spectral properties that is only based on parametric resonance between weak SPP field and directional SSPP, which demonstrates the technical novelty of this work. Moreover, our proposal introduces the quantum properties of the atomic medium such as superradiant SPP propagation, and quantum dephasing suppression to produce a coherent intense plasmonic field, which conceptually is different from other works, which are mainly based on semi-classical treatment^20^ and hence is novel. This amplification scheme, consequently, is quite different from previous methods and we provide detailed steps towards this novel plasmonic lasing scheme.

Our method for generating intense SPP field using a weak plasmonic field is based on three steps: (i) launching directional SSPP, (ii) providing parametric resonances and field amplification, and (iii) detection and stability of the output beam. To justify these steps, first, we elucidate the basic concepts of our idea in “Background”. Next, we underpin our model and elucidate its feasibility, from source to detection, in an experiment by proposing realistic source-waveguide-detection triplet in “Model”. Then we present our results in three parts: first we elucidate our protocol for exciting SSPP in “Superradiant surface-plasmon polariton launching”, next we elucidate weak field amplification and its stability in “Surface plasmon polariton amplification”, and finally we suggest the detection system in “Detection of amplified plasmonic field”. We discuss the possible applications such as nanolaser, plasmonic field effect transistor in “Discussion” and finally we summarize our work in “Conclusion”. In this work, we qualitatively describe the system within the body of the paper and we present detailed quantitative and mathematical steps within supplementary material.

## Background

Inversion-less lasing and coherent amplification of SPP waves need three concepts that are intensively investigate in previous studies: (i) *superradiant emission of radiation*, (ii) *parametric resonance*, and (iii) *weak field amplification and stability analysis*. Consequently, we begin this section by briefly reviewing the superradiant emission of radiation in “Superradiant emission of radiation”. Next, we introduce the parametric resonance and discuss the possibility of providing gain for a weak driving field “Parametric resonance”. Finally, we review the salient aspect of the weak field amplification using the Mathieu equation. This concept is discussed in “Weak field amplification and dynamical stability”.

### Superradiant emission of radiation

In this subsection, we elucidate the pertinent concept of the superradiant emission of radiation^36–38^. For a *N* two-level atoms with ground state $| {b} \rangle$ and excited state $| {a} \rangle$ that are situated within a cell of radius *R* much smaller than the radiation wavelength $\lambda$, a uniform absorption of photon by a single quantum emitter prepares the atomic ensemble to the so called Dicke-state

1 $$\begin{aligned} | {\Psi _\text {s}} \rangle =\frac{1}{\sqrt{N}}\sum _{j=1}^{N} | {b_1,b_2,\ldots ,a_j,\ldots ,b_N} \rangle . \end{aligned}$$

This collectively excited atomic state decays into $| {b_1,b_2,\ldots ,b_N} \rangle$ at a rate $\Gamma _\text {s}=N\gamma$; $\gamma$ is the single-atom decay rate, and consequently produce a superradiant emission^33^. On the other hand, wavevector of the propagated photon ($k_\text {a}$) would record through time-Dicke state

2 $$\begin{aligned} | {\psi _\text {a}} \rangle =\frac{1}{\sqrt{N}}\sum _{j=1}^{N}\exp \{\text {i}\varvec{k}_\text {a}\cdot \varvec{r}_{j}\} | {b_1,b_2,\ldots ,a_j,\ldots ,b_N} \rangle , \end{aligned}$$

if this single photon is uniformly absorbed by an atom situated on $\varvec{r}_{j}$ within the atomic ensemble^39^. Consequently, an atomic medium prepared to a time-Dicke state and situated above a metallic like layer, may produce a surface polaritonic superradiant emission through spontaneous decay to ground state and thereby produces a photon with wavevector $\varvec{k}_\text {a}$ and energy $E=\hbar \omega _\text {ab}$^16^. In our work, directional SSPP launches for the wavenumber $\varvec{k}_\text {SPP}=\varvec{k}_\text {a}$ and perturbation frequency $\omega _\text {SPP}=\omega _\text {ab}$.

### Parametric resonance

We start this subsection by introducing the concept of parametric resonance^40,41^. Parametric resonance is known as a process in which the parameters that describe a system possesses time variation or temporal evolution. In a physical configuration that is characterized with a periodic system parameter *a*(*t*), and its dynamical evolution can be described with the Hill’s differential equation^42^ the parametric resonance occurs if $a(t)=a(t+m'T)$, for any positive integer $m'\in \{2,3,4,\ldots \}$. It follows from Floquet’s theorem^43,44^ that the Hill’s equation with a periodicity factor $\sigma$ has an arbitrary solution *X*(*t*) such that $X(t+2\pi )=\sigma X(t)$. The periodicity factor depends on the system parameters (see Supplementary Information S8.1 of the supplementary materials for more details.).

In this work, we employ the concept of parametric resonance to establish weak plasmonic field amplification in the presence of SSPP radiation. This amplification is achieved for a characteristic frequency $\omega _\text {ch}$ satisfying $\omega _\text {ch}=\omega _0/m$, for $\omega _0$ the frequency perturbation of the plasmonic field in which the amplification occurs.

### Weak field amplification and dynamical stability

This subsection describes the key concepts of the weak field amplification and discusses the stability of the amplified field. Amplitude enhancement and amplification can be achieved within a dynamical system by exploiting *forced* oscillations^45^ and *parametric* oscillations^46^. In a physical system with two control parameter *a*, *q* whose temporal dynamics is described with a specific form of Hill’s equation known as Mathieu equation parametric resonances would yield the field amplification (see Supplementary Information S8.1 for mathematical details towards amplification process). We notice that the strongest amplification is achieved for the first order of resonance ($m=1$)^33^.

We achieve the solution of the Mathieu’s equation in terms of arbitrary constants *A*, *B*, periodicity exponent $\mu$ and a periodic function $\Phi (t)$ by exploiting the Floquet’s theorem $X(t)=A\exp \{\mu t\}\Phi (t)+B\exp \{-\mu t\}\Phi (-t)$, that establishes stable amplification only for the specific values of *a* and *q*^47^. In this work, we establish that the dynamical evolution of the stable weak plasmonic field in the presence of SSPP describes by Mathieu-like equation that can be amplified through parametric resonances.

*Possible waveguide configuration* Following these concepts, to achieve inversion-less coherent SPP beam, a directional superradiant SPP is needed to provide characteristic frequency, a weak signal field that is in resonance with this superradiant pulse then irradiates the medium and parametric oscillation of this weak field in the presence of superradiant emission would yield weak field amplification. Consequently, our apparatus should comprises modified laser fields coupled to atomic medium-metallic like interface to produce plasmonic superradiance, and we need a weak signal field to amplify through parametric oscillation.

## Model

Based on aforementioned justification, we qualitatively describe the plasmonic configuration, which comprises three parts, (i) source, (ii) waveguide and (iii) detection. Consequently, we begin our description by elucidate laser fields as source in “Source”, then we explain the waveguide configuration by describing the metamaterial layer and atomic medium in “Waveguide” and finally we describe the detection system for measuring the output spectral intensity of weak plasmonic field in “Detection”. Furthermore, we briefly discuss the possible realistic model of source-waveguide-detection triplet in “Possible realistic model”.

### Source

The generation and amplification of the coherent SSPP field are obtained by exploiting four fields: a weak signal (s), a strong driving (d), a couple (c) fields and an incoherent flash lamp. We assume our fields all share the same polarisation and are obtained from a dye laser that is frequency stabilized, linearly polarised, possesses enough spatial coherence to cover the waveguide and temporally longer than amplification scale^48^. Acousto-optic modulators control the carrier frequency of each beam. The fourth driving field is an optical pump from a flash lamp with long temporal width and linearly polarised, and spatially broadened enough to cover all the interaction surface^16^.

### Waveguide

Now we elucidate our plasmonic configuration. Laser fields as sources are injected on our planar waveguide, which comprises three layers: a substrate that a metamaterial can build on it, a double-negative-index metamaterial as middle layer and a dielectric on the top (see Fig. 1). On one ends of the waveguide an optical fiber is attached^49^ and, on the other end, a Bragg grating structure^50^. The top of the NIMM layer, which could be constructed as a nano-fishnet structure^51^, is doped by atoms or molecules serving as electric dipoles, and the depth of this dopant layer is a few dipole resonant wavelengths. The flash-light irradiates through the dielectric into the waveguide normal to the interface, signal and driving lasers are injected and co-propagate parallel to the interface with the end-fire coupling technique^52^. The couple field is separated into the two contra-propagate fields and introduced to the waveguide at a small angle normal to the interface. This source-waveguide-detector triplet is experimentally feasible and efficient for quantum SPP excitation^53^.

### Detection

Finally, we explain the operation of laser fields and explain our proposed detection scheme. The optical pump is applied to induce collective excitation of the atomic medium^54,55^, the couple field provides an opportunity to generate directional SSPP^56^, the strong driving field introduces a quantum gain to the hybrid plasmonic structure^33^, and the signal field produces a weak SPP field that we are supposed to amplify. These polaritonic waves propagate into the Bragg regime. This Bragg structure is a dielectric with modified optical properties. We employ a tapered multimode optical fiber due to its efficiency for detecting the intensity of the amplified SSPP. This fiber is suspended above the Bragg regime, which presumably is evanescently coupled to the Bragg regime of the waveguide. Certain spectral components are preferentially scattered. Spectral properties of the field propagating through the fiber are measured and used to infer spectral properties of the surface plasmon polariton.

### Possible realistic model

Specifically, we assume $\text {Pr}^{3+}$ ions doped in $\text {Y}_{2}\text {Si}\text {O}_{5}$ crystal with corresponding atomic levels $| {1} \rangle = | {^2\text {H}_4,F=\pm (5/2)} \rangle$, $| {2} \rangle = | {^3\text {P}_0} \rangle$ and $| {3} \rangle = | {^2\text {D}_1,F=\pm (1/2)} \rangle$. This medium has atomic density $N_\text {a}$, the natural decay rate for $| {m} \rangle \leftrightarrow | {n} \rangle$ is $\Gamma _{mn}$, dephasing rate is $\gamma _{mn}^{\text {deph}}$ and the scheme is also cooled up to $4~\text {K}$ and inhomogeneously broadened by $W_{ij}$^57^ that affects the SSPP amplification. However, we consider spectral hole burning technique^58^, thereby minimize the effect of these broadening on generated SPP and dynamics of weak SSPP.

Our waveguide configuration is a multilayer structure comprised of an atomic medium and a metallic layer. The atomic medium appeals due to its efficiency for providing directional superradiant emission of radiation and we consider metallic-like to produce the SPP field. To increase the amplification efficiency of the weak SPP field, this metallic layer should be ultra-low loss within the surface polaritonic transition frequency, which is in the optical frequency region for our proposed atomic medium. Double-negative-index metamaterials with nanofishnet structure operate within the optical frequency range and possess ultra-low Ohmic loss for the SPPs in this spectral range, thus this structure is suitable to exploit as a metallic-like layer within the waveguide configuration.

There are different metamaterial layers operate in the optical frequency region^51,59^. Our suggested NIMM layer is a nano-fishnet metamaterial. Specifically, we consider $\text {Al}_2\text {O}_3$–$\text {Ag}$–$\text {Al}_2\text {O}_3$ multi-layer with rectangular nano-holes as our NIMM, which possesses low Ohmic loss for the SPPs within optical frequencies^60,61^ that can be effectively reduced and suppressed. To this aim, various mechanism such as optical parametric amplification^62^, geometrical tailoring and optimization^63^, including gain media^64^ and meta-surfaces^65^ serve to combat the Ohmic loss related to plasmonic structures, specifically for optical frequencies. However, we introduce a *virtual* gain to our hybrid interface by employing a coherent method, which is based on constructive interference of the two externally injected plasmonic fields and also excited signal SPP field to suppress the Ohmic loss of this NIMM layer^66,67^. This loss-compensation is basically different from the stimulated optical loss suppression achieved by dye molecules or amplification induced in integrated plasmonic chip^64,68^.

**Figure 2:**
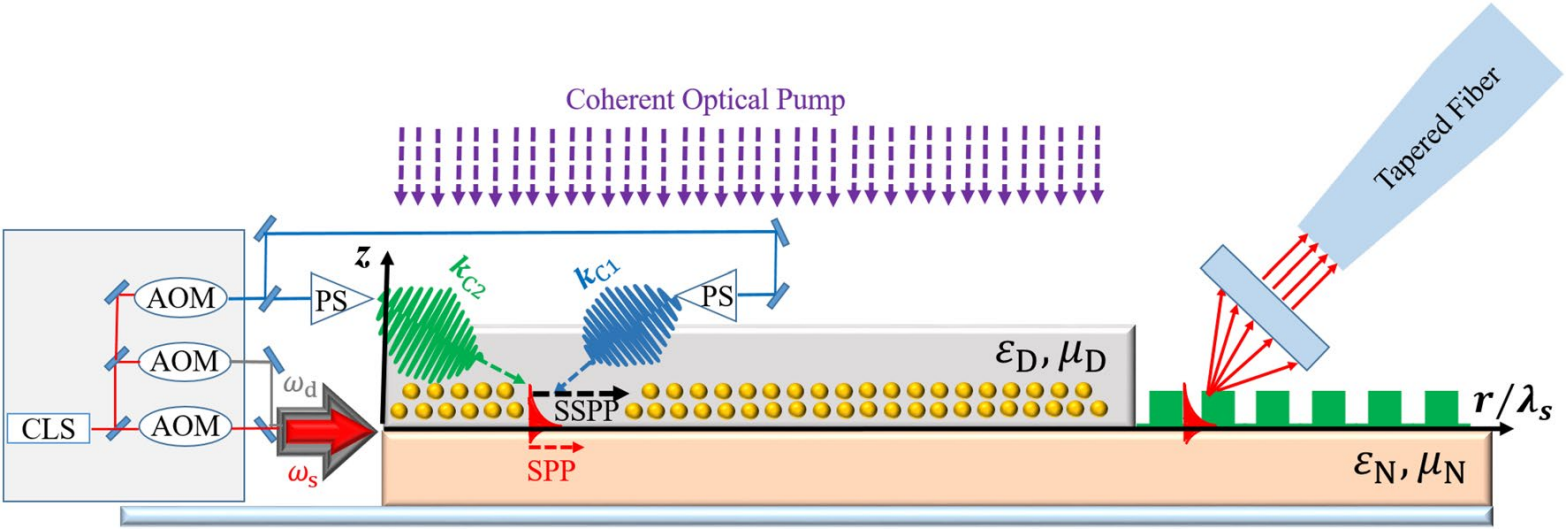
Possible experimental realization of the waveguide: This figure comprises plasmonic waveguides, tapered optical fiber, Bragg grating and lasers that represents our proposed source-waveguide-detection triplet. Yellow dots are the $\text {Pr}^{3+}$ ions within the $\text {Y}_2\text {SiO}_5$ crystal, which represents our atomic medium. Here, longitudinal axis is described and normalized $r/\lambda _{s}$ which characterized the direction of the SSPP emission, and we consider the *z* axis as transverse coordinate. The green lattice at the end of the waveguide represents the schematic of Bragg grating. In this figure we have employed some abbreviations as follows. *CLS* Coherent laser source, *AOM* acousto-optic modulator, *PS* Pulse shaper.

*Vision for possible realistic model* Now we elucidate our proposed source-waveguide-detection triplet in details, as it is shown in Fig. 2. The frequency of the laser sources are finely tuned by acousto-optic modulator and then enter the interface using a coupler. The directional SSPP then excites in the characteristic direction $\varvec{r}$ for $I_\text {s}=I_\text {d}=0$ due to spontaneous emission of the $| {3} \rangle \leftrightarrow | {1} \rangle$ transition with $\lambda _\text {SSPP}=\lambda _{31}$ through coupling atomic superradiant to the interaction interface. Next, the signal laser irradiates the interaction interface and induce a weak SPP field with central frequency $\omega _\text {s}$ and we introduce another driving laser to suppress the unwanted Stark effect. The image of the excited SPP field through signal field illumination is represented as an evanescence field characterized by $\varvec{E}_\text {s}(r,z)$ in the Fig. 2. Consequently, the SPP wavelength is the same as confined signal field wavelength $\lambda _\text {s}=\lambda _\text {SPP}$, and we define this wavelength as a characteristic length-scale of the waveguide. Based on this discussion, in Fig. 2 we normalize the longitudinal axis in terms of $r/\lambda _\text {s}$. It is obvious from this figure that the waveguide dimension should be a few times larger than our proposed characteristic length. In this work, we achieve this amplification for waveguide size $|\varvec{r}|\approx \;200~\upmu \text {m}$ and $\lambda _\text {s}\approx \;607~\text {nm}$. In the longitudinal axis in Fig. 2 we depict the ratio $r/\lambda _\text {s}$ to represents the approximate size of the waveguide compared to dipole transition wavelength. The output signal propagates to a Bragg grating and the scattered intensity of the SPP field is then collected using an image intensifier, and this collector coupled this scattered light to a multimode tapered fiber.

## Results

We present the main results of this paper in four sections: First, we give a qualitative description of directional SSPP propagation and directional SPP lasing operation in “Brief description of SSPP launching and SPP lasing operation”. Second, in “Superradiant surface-plasmon polariton launching” we extend the plasmonic superradiant emission and discuss the directional launching of SSPP. Next, “Surface plasmon polariton amplification” we present the details of weak SPP field amplification in the presence of this directional plasmonic superradiant radiation. Finally in “Detection of amplified plasmonic field” we suggest a technique to detect directional amplification of surface polaritonic wave.

### Brief description of SSPP launching and SPP lasing operation

Stable propagation of SPP waves and robust directional launching of SPP are sufficient conditions to achieve efficient lasing of SPP waves without need to population inversion. However. our hybrid plasmonic interface is inherently dissipative, and consequently propagation length and stability of the excited SSPP would be highly limited due to high loss. Here we suppress the Ohmic loss related to the NIMM layer by inducing the concept of virtual gain that provides stable propagation of SPP waves and robust launching of directional SSPP. This configuration then is suitable for both directional SSPP launching and stable propagation of SPP field, and provides opportunities to coherent amplification of weak plasmonic field by generating polaritonic superradiant. In order to achieve this coherent amplification, first, we discuss directional plasmonic superradiant excitation and then we propose amplification of the weak SPP field. In what follows, we qualitatively elucidate the main steps towards launching SSPP excitation, then as a second step we give a brief discussion on weak plasmonic field amplification.

*Launching directional SSPP* Directional SSPP within interaction interface is launched by collective excitation of atomic ensemble through two steps. First we perpendicularly illuminate the interaction interface using an optical pump with intensity $I_\text {p}$ to excite a single atom through coupling with $| {1} \rangle \leftrightarrow | {2} \rangle$ transition. Second, the contra-propagating couple laser fields with $(2n_\text {p}+1)\pi$ pulses and alternating wavevectors $\varvec{k}_{\text {C}\imath }$; $\imath \in \{1,2\}$ drive the $| {2} \rangle \leftrightarrow | {3} \rangle$ atomic transition. The spontaneous emission of $| {3} \rangle \leftrightarrow | {1} \rangle$ transition for the atom in position $\varvec{r}_{j}$ consequently generates a single SPP mode with characteristic wavenumber^16^

3 $$\begin{aligned} \varvec{k}_\text {SPP}=\left( n_\text {p}+1\right) \varvec{k}_{\text {C}2}-n_\text {p}\varvec{k}_{\text {C}1}, \end{aligned}$$

and this directional plasmonic emission serves as directional superradiant mode due to the atomic medium being prepared as^39,69^

4 $$\begin{aligned} | {\psi _\text {SPP}} \rangle =\frac{1}{\sqrt{N_\text {a}}} \sum _{i=1}^{N_\text {a}}\exp \left[ \text {i} \varvec{k}_\text {SPP}\cdot \varvec{r}_i\right] | {3_{i}} \rangle \otimes _{j\ne i} | {1_{j}} \rangle . \end{aligned}$$

Our proposed Dicke-state with wavenumber $k_\text {SPP}$ and frequency $\omega _\text {SPP}$ decays faster than the single atom spontaneous emission, thereby acts as directional superradiant emission^33,39^.

*Weak plasmonic field amplification* We describe the coherent amplification of the weak SPP wave in five steps. First, we employ Schrödinger equation formalism to introduce a directional SSPP emission between $| {3} \rangle \leftrightarrow | {1} \rangle$ transition^39^ within the atomic medium-NIMM layer interface. Second, we assume the weak signal SPP field strongly coupled to the interface with a evanescence function, propagate as a traveling wave and possesses a constant phase whose spatiotemporal dynamics is achieved by using the coupled Maxwell–Schrödinger commensurate with its Fourier spectrum. Next, we employ a coherent loss-compensation mechanism and quantum decoherence suppression by using the resonant coupling between the collective atomic excitation and plasmonic fields. Finally, we investigate the coherent amplification commensurate with stability analysis of this weak plasmonic wave by introducing the parametric resonance to this interface. Consequently, to quantitative description of the system, we employ three well-established assumptions, namely (i) Schrödinger equation to launch directional SSPP, (ii) Drude–Lorentz model to describe nano-fishnet NIMM layer, and (iii) Maxwell–Schrödinger equation to achieve spatiotemporal dynamics of weak signal SPP.

*Vision for description of the plasmonic system* We express our results by presenting qualitative description and physical explanation towards weak plasmonic field amplification and present mathematical steps and quantitative descriptions within the “Fourier optics of surface plasmons”. Our qualitative description is based on spatial and spectral evolution of the weak SPP field in the presence of directional superradiant radiation, while our quantitative description for obtaining weak SPP amplification is based on three main equations, namely, (i) dynamics of the excited atomic state, Eq. (7), (ii) dynamics of the weak SPP field in the interaction interface, Eq. (10), and (iii) weak-field amplification through Mathieu-like equation Eq. (18).

### Superradiant surface-plasmon polariton launching

In this section, we elucidate our method towards launching directional plasmonic superradiant emission that excites and propagates as a quantum plasmonic mode^16^. We achieve SSPP by preparing the atomic medium to a time-Dicke state characterized by Eq. (4). To efficient excitation of SSPP, we suggest the heralded atomic ensemble^70^ due to its efficiency for generating superradiant pulse. To investigate directionality and robust launching of SSPP, we also consider a simple case where the driving (d) and signal (s) fields are switched off ($\Omega _\text {d}=\Omega _\text {s}=0$). Consequently, coupling optical pump and $(2n_\text {p}+1)\pi$ couple pulses as we describe in “Brief description of SSPP launching and SPP lasing operation” would yield directional and robust excitation of SSPP.

We describe this quantum plasmonic excitation in our dispersive and dissipative interface by exploiting a canonical quantization method^71^ through introducing a quantized plasmonic electric field $\hat{\varvec{E}}$, and a quantized current density $\hat{\varvec{j}}$. This quantized field is characterized with a Green tensor $\mathcal {A}_\text {SPP}$ that we achieve for our hybrid dielectric-metamaterial interface through using Fourier optics of SPP waves as represented in Ref.^72^. The quantum plasmonic field describes by $\hat{\varvec{C}}_{\jmath }(\varvec{r},\omega )$ ($\hat{\varvec{C}}_{\jmath }^{\dagger }(\varvec{r},\omega ))$; $\jmath \in \{\text {e},\text {m}\}$ as annihilation (creation) operators associated with the electrical (e) and magnetic (m) response of the medium, whose components are described by usual bosonic commutation relation $[C_{\jmath i}(\varvec{r},\omega ),C_{\jmath ' j}(\varvec{r}',\omega ')]=0$ and $[C_{\jmath i}(\varvec{r},\omega ),C_{\jmath ' j}^{\dagger }(\varvec{r}',\omega ')]=\delta _{ij}\delta _{\jmath \jmath '}\delta (\omega -\omega ')\delta (\varvec{r}-\varvec{r}')$. Finally, this plasmonic field is coupled to the atomic medium whose dynamical evolution is achieved using atomic Pauli matrices $\sigma _{l}^{z}= | {3_{l}} \rangle \langle {3_{l}} |- | {1_{l}} \rangle \langle {1_{l}} |$ and $\sigma _{l}^{x}= | {3_{l}} \rangle \langle {1_{l}} |+ | {1_{l}} \rangle \langle {3_{l}} |$. Considering the dipole moment of the system as $\varvec{d}_{l}$ the Hamiltonian of this hybrid interface becomes (see “1.1” for the details of derivation)

5 $$\begin{aligned} H=&\sum _{l=1}^{N_\text {a}}\left[ \frac{\hbar \omega }{2}\sigma _{l}^{z}-\sigma _{l}^{x}\varvec{d}_{l}\cdot \varvec{E}_{l}(\varvec{r}_{l})\right] \nonumber \\&+\sum _{\jmath }\int \text {d}^3\varvec{r}\int _{0}^{\infty }\text {d}\tilde{\omega }\hbar \tilde{\omega }\hat{\varvec{C}}_{\jmath }^{\dagger }(\varvec{r}',\tilde{\omega })\cdot \hat{\varvec{C}}_{\jmath }(\varvec{r}',\tilde{\omega }). \end{aligned}$$

Consequently, the quantum plasmonic field launches as superradiant SPP for atomic medium prepared at Eq. (4) and we employ the Schrödinger equation $\partial | {\Psi (t)} \rangle /\partial t=-(\text {i}/\hbar )H | {\Psi (t)} \rangle$ to evaluate the dynamics of the SSPP. To this aim, the time dependent amplitude transition for the $| {j} \rangle$ atomic transition is $c_{j}(t)$, $| {\phi } \rangle = | {g_1,g_2,\ldots ,g_N} \rangle$ is the ground state and $| {\varvec{I}_{\jmath ,m}} \rangle =\hat{C}_{\jmath m}^{\dagger }(\varvec{r}',\omega ) | {\phi } \rangle$ denotes the excited plasmonic mode. Therefore the evolution of the SSPP would then achieve by considering the atomic transition at $t=0$ as Eq. (4), assume Ansatz

6 $$\begin{aligned} | {\Psi (t)} \rangle =&\sum _{l=1}^{N_\text {a}}c_{3,l} | {3_{l},\phi } \rangle \otimes _{j\ne l} | {1_{j}} \rangle \nonumber \\&+\sum _{\jmath ,m}\int \text {d}^3\varvec{r}\int _{0}^{\infty }\text {d}\tilde{\omega }c_{1j}(\tilde{\omega },\varvec{r}') | {g,\varvec{I}_{\jmath , m}} \rangle , \end{aligned}$$

and solving Schrödingr equation commensurate with Eq. (5).

To achieve the equation of motion, we define the emitter-emitter coupling between the two characterized atoms (*a*, *b*) as $(\mu _0\omega ^2/\pi )\varvec{d}_\text {a}\cdot \mathcal {A}_\text {SPP}\cdot \varvec{d}_\text {b}\sim g_\text {ab}$ and consider the dissipation of the interaction interface as $\xi (\varvec{q}):=\langle {\varvec{q}|\varvec{L}} \rangle :=\text {e}^{\text {i}\varvec{q}\cdot \varvec{L}}$. Next, we calculate the emitter–emitter coupling in the Fourier-space $\omega -\varvec{k}_{\parallel }$ by considering $g(\varvec{k}_{\parallel })$ as Lorentzian line shape. Finally, solving the Schrödinger equation for *j*th excited atomic states situated at $\varvec{r}_{j}$ would then yield integro-differential equation

7 $$\begin{aligned} -\frac{\partial c_{3}(t)}{\partial t} =\omega _\text {o}^2\exp \left\{ \left( t_\text {L}\gamma _\text {SPP}\right) ^2\right\} \int _0^{t}\text {d}t'\;K(t,t')c_{3}(t'), \end{aligned}$$

with $t_L=L/v_\text {SPP}$ the SPP-flight time, $\tau _\text {l}=\gamma _\text {SPP}^{-1}$ is the time related to loss, with $\omega _\text {o}^2:=[N_\text {a}/(2L)^2]g_{ab}$, the oscillation frequency of quantum SPP and with

8 $$\begin{aligned} K(t,t')=\exp \left\{ \frac{-(t-t'+2t_L^2\gamma _\text {SPP})^2}{(2t_L)^2}\right\} , \end{aligned}$$

the kernel of (7) (see Supplementary Information S8.1 for mathematical details towards derivation of Eq. (7)). This equation is a boundary value problem with certain initial condition. In our configuration, we achieve plasmonic superradiant emission by preparing the atomic medium to a time-Dicke state. Consequently, in solving Eq. (7) we assume $c_{3}(t=0)=1$ and we neglect the temporal evolution of this excited state.

**Figure 3:**
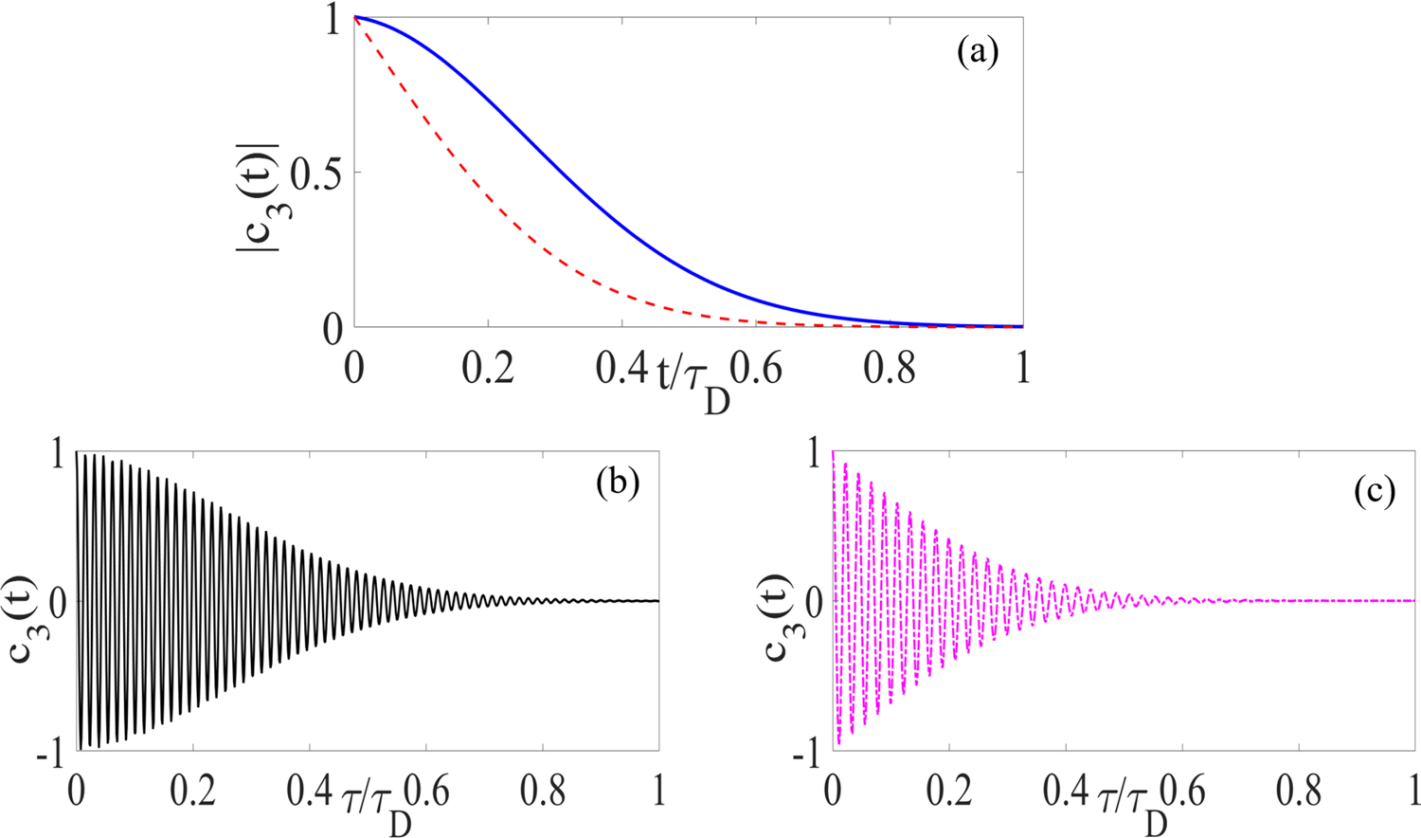
Panel (**a**) denotes the bound solution of our integrodifferential equation. Panels (**b**) and (**c**) represent the numeric solution of the Eq. (7). In both panels, the solid lines (blue and black) represent the dynamics of the $| {3} \rangle$ states for NIMM layer with coherent loss-suppression, while the dashed-line (red and magenta) curves are the results with the simple nano-fishnet metamaterial structure.

Now, we investigate the dynamical evolution of the atomic states in Fig. 3. The dynamics of the atomic ensemble through the time-Dicke state and the resultant collective oscillation would directly proportional to the SSPP emission in the Fourier domain. The spontaneous emission of this atomic medium provides a SSPP radiation with a direction satisfying Eq. (3). The temporal evolution of excited atomic state (7) is a Gaussian profile with fast oscillations that appear as the absorption re-emission of the quantum plasmon is faster than the total plasmonic loss. These damped oscillations also depend on propagation time ($t_\text {SPP}$), oscillation frequency ($\omega _\text {o}$) and total Ohmic loss of the system as it is shown in Fig. 3b,c. The Fourier component of this spontaneous emission is a Lorentzian line-shape with a sharp maximum in $\varvec{q}\approx \varvec{k}_\text {SPP}$, which guarantees this spontaneous emission propagates as directional SSPP. This oscillation regime can be controlled through our coherent loss-compensation scheme and is in agreement with previous studies on SSPP dynamics within dissipative interface^16^.

Our waveguide thereby acts as a high-speed single-photon switch. This switching is expected by considering excited atomic-state dynamics as $c_{3}(t)=|c_{3}(t)|\exp \{(\text {i}\omega _\text {o}-\gamma _\text {SPP})t\}$ (for $\gamma _\text {SPP}$ the total decay rate of the system). The temporal dynamics represents the strong coupling between the directional SPP and atomic medium. We also introduce resonant coupling between the directional SSPP mode and two-externally coupled plasmonic field to coherent compensation of metamaterial loss $\text {Im}[\varepsilon _\text {N}]\approx 0.2~\text {cm}^{-1}$, and modulate the field dispersion $\text {Re}~(1/k_\text {N})\approx 200$, yielding modified dephasing time. This SSPP dynamics is then fast, low-loss, and preserves coherence due to efficient quantum decoherence suppression^4^, which can be exploited to design coherent single-photon switch. Our plasmonic configuration therefore excites directional superradiant polaritonic field and consequently, the weak plasmonic field within this waveguide can amplify without need to population inversion.

Now, we investigate the robustness of this superradiant emission, against experimental imperfections. Our plasmonic scheme, exhibits superradiant plasmonic radiation only if the waveguide decay ($\Gamma _\text {W}$) exceed free space decay ($\Gamma _\text {F}$), $\Gamma _\text {W}\gg \Gamma _\text {F}$. We consider a specific case of $N_\text {a}$ quantum emitter and *m* collective atomic excitation with $m\ll N_\text {a}$ that can decay into *m*-photon wave packet with error scale $\varepsilon _\text {lin}\sim m\Gamma _\text {F}/(N_\text {a}\Gamma _\text {W})$^73,74^. Here, we exploit an experimentally feasible heralded scheme, and for $\Gamma _\text {W}\approx 5\times 10^{-3}\Gamma _\text {F}$ and for collective excitation rate $m/N_\text {a}\sim 10^{-2}$, our system launches directional SSPP with an error $\varepsilon _\text {lin}\approx 10^{-5}$. Consequently, our suggested scheme is robust against photon loss in the limiting case of $m\ll N_\text {a}$ and thus provides opportunity for weak SPP field amplification. In “Surface plasmon polariton amplification” we establish coherent amplification of the weak SPP field in the presence of this directional SSPP radiation.

### Surface plasmon polariton amplification

Now, we elucidate the possibility of weak SPP amplification and lasing without the need for population inversion in our waveguide and investigate the stability of our field amplification. Consequently, in “Surface plasmon polariton amplification” we describe the necessary quantitative steps for weak SPP field amplification (we left the detailed mathematical description to Supplementary Information S10) and then in “Surface plasmon polariton amplification” we represent our qualitative description towards SPP field amplification and investigate the stability of this lasing process.

#### Dynamical evolution of the weak SPP field within interaction interface: Maxwell–Schrödinger equation

In this section, we extend the concept developed earlier for the case that both signal and driving field are coupled to the interaction interface. We establish that this plasmonic system generates coherent SPP amplification without the need for population inversion. These driving and signal fields have Rabi frequencies $\Omega _\text {d}$ and  $\Omega _\text {s}$, respectively, that are tightly confined to the interface by transversely evanescence coupling functions $\zeta _\text {d}(z)$ and $\zeta _\text {s}(z)$^6,29^, through $\Omega _{m}:=\zeta _{m}(z)\Omega _{m}; m\in \{\text {d},\text {s}\}$ and we take into account the plasmonic evanescence coupling by employing field averaging technique (i.e. $\langle {\zeta (z)\Omega _l} \rangle \mapsto \Omega _l$^29^).

Next, we consider the case where a weak signal field and a strong driving field are injected to the system as SPP modes. We treat the signal plasmonic field as a plane wave with group velocity $\varvec{v}_\text {SPP}=[\partial \varvec{k}_\text {SPP}/\partial \omega ]^{-1}$ for $\varvec{k}_\text {SPP}$ the linear dispersion of the plasmonic mode. The perturbation frequency is $\omega$ and its wavenumber is $\varvec{q}$. To achieve the weak plasmonic field dynamics, we employ Green function approach^72^ by introducing a dyadic Green tensor $\varvec{\tilde{\mathcal {A}}}_{13}$ that depends on the optical properties of the interaction interface. The Fourier spectrum of the weak plasmonic wave is then

9 $$\begin{aligned} \tilde{\Omega }_\text {s}\approx \int \;\frac{\text {d}^2\varvec{q}}{(2\pi )^2}\varvec{\tilde{\mathcal {A}}}_{13}\hat{j}\text {e}^{\text {i}[(\varvec{q}-\varvec{k}_\text {SPP})\cdot \varvec{r}-\omega t]}. \end{aligned}$$

The plasmonic field are highly dissipative for $\varvec{q}\gg \varvec{k}_\text {SPP}$ and $\omega \gg \omega _\text {SPP}$ due to Ohmic loss and atomic medium absorption. Therefore we assume a small deviation $\varvec{q}\approx \varvec{k}_\text {SPP}+\mathcal {O}(\varvec{q-\varvec{k}_\text {SPP}})$ and $\omega =\omega _\text {SPP}+v_\text {SPP}\delta \omega +\mathcal {O}(\delta \omega ^{2})$ to this perturbation parameters^5,29^. The plasmonic mode will stably propagate within interface that its optical properties are described by macroscopic Drude-Lorentz model^28,75^.

Our approach for obtaining the weak plasmonic field dynamics is then based on Fourier analysis method developed in Ref.^72^, which incorporates the dissipation and dispersion of the SPP waves in a phase space characterized by real wavenumber ($\varvec{q}$) and complex perturbation frequency ($\tilde{\omega }$) (we elucidate this method in “Fourier optics of surface plasmons”). Finally, we achieve the reduced Maxwell equation for this weak signal field within our characteristic $\tilde{\omega }$-$\varvec{q}$ space by considering the stable propagation regime, taking derivatives with respect to *t* and $\varvec{r}$ as

10 $$\begin{aligned} \left( \frac{\partial }{\partial t}+\varvec{v}_\text {SPP}\cdot \varvec{\nabla }\right) \Omega _\text {s}=\text {i}\mathcal {C}\tilde{\rho }_{31}, \end{aligned}$$

with $\mathcal {C}=N_\text {a}(\pi \mathcal {A}_\text {SPP})/(\gamma _\text {SPP})$. Note that our Eq. (10) is similar to Maxwell–Schrödinger equations obtained in earlier works^33^, however (10) differs from previous works due to incorporating dissipation and dispersion of the surface-plasmon mode to the atomic medium evolution. We note that the dipole moment of the system would be proportional to the $| {3} \rangle \leftrightarrow | {1} \rangle$ atomic transition which is characterized in Eq. (10) by $\tilde{\rho }_{31}$. This term in (10) is atomic coherence term, which is related to atomic transition amplitudes as

11 $$\begin{aligned} \tilde{\rho }_{31}=c_{3}(t)c_{1}^{*}(t)\text {e}^{-\text {i}\varphi -\gamma _\text {SPP}t}. \end{aligned}$$

Equation (10) commensurate with coherence term (11) describes the dynamics of the weak signal field within this dissipative interface. In the next section we discuss the amplification condition and establish that this weak plasmonic field can be amplified for specific modulation of the driving field.

In order to evaluate the temporal evolution of the weak signal field, we consider $\zeta _\text {s}(z)\approx \zeta _\text {d}(z):=\zeta (z)$. The atomic states within the interaction interface then coupled to the weak signal field that are modulated as

12 $$\begin{aligned} \Omega _\text {s}&=\zeta (z)\Omega _\text {s}(t)\exp \{\text {i}\varphi \},\nonumber \\ c_{\imath ,\text {s}}&=\zeta (z)c_{\imath ,\text {s}}(t)\exp \{\text {i}\varphi \}+\text {c}.\text {c}, \end{aligned}$$

for $\varphi :=\tilde{\omega }_\text {SPP}(t-x/v_\text {SPP})$ the phase of the plasmonic wave.

Moreover, to efficient amplification of this weak plasmonic field, we consider that the deriving field is circularly polarized as $\Omega _\text {d}(x,t)=\bar{\Omega }_\text {d}^{(1)}(x,t)\hat{\epsilon }_{+}+\bar{\Omega }_\text {d}^{(2)}(x,t)\hat{\epsilon }_{-}$; $\epsilon _{\pm }=(\varvec{e}_{x}\pm \text {i}\varvec{e}_{y})/\sqrt{2}$ represents right (+) and left (-) circular polarizations, respectively. This polarized field with Rabi frequencies  ($\Omega _\text {d}^{(1)},\Omega _\text {d}^{(2)}$) components couples the ground atomic state to an intermediate atomic transition $| {a} \rangle$ through $| {1} \rangle \leftrightarrow | {a} \rangle$ state and the signal field excites the $| {1} \rangle \leftrightarrow | {3} \rangle$ transition, as clearly shown in Fig. 1. To achieve the dynamics of this hybrid scheme, we consider the Hamiltonian of the system (see Eq. (S58) of supplementary material), and employ the Schrödinger approach. Next, we evaluate the temporal evolution of the atomic medium and consequently achieve the coherent atomic term characterized by Eq. (11). Then we replace the coherence term in Eq. (10), and take the derivative with respect to time. Finally, after some mathematical steps represented in Supplementary Information S10.2 of supplementary material, we achieve the dynamical evolution of this weak signal field as

13 $$\begin{aligned} \left( \frac{\partial }{\partial t}+\beta (x,t)\right) \left( \frac{\partial }{\partial t}+\varvec{v}_\text {SPP}\cdot \varvec{\nabla }\right) \Omega _\text {s}=\text {i}\mathcal {C} f(x,t)\Omega _\text {s}, \end{aligned}$$

here the coefficients $\beta$ and *f* are

14 $$\begin{aligned} \beta :=&\tilde{\Delta }+\frac{\bar{\Omega }_\text {d}^{(1)2}+\bar{\Omega }_\text {d}^{(2)2}}{-\text {i}\omega _\text {SPP}+\gamma _\text {SPP}}+\frac{4\bar{\Omega }_\text {d}^{(1)}\bar{\Omega }_\text {d}^{(2)*}\cos (2\varphi )}{\omega _\text {SPP}+\text {i}\gamma _\text {SPP}},\nonumber \\ f:=&1-\frac{2\bar{\Omega }_\text {d}^{(1)2}}{\tilde{\Delta }(-\text {i}\omega _\text {SPP}+\gamma _\text {SPP})}-\frac{2\bar{\Omega }_\text {d}^{(1)2}+\bar{\Omega }_\text {d}^{(2)2}\exp \{2\text {i}\varphi \}}{\omega _\text {SPP}(-\text {i}\omega _\text {SPP}+\gamma _\text {SPP})}. \end{aligned}$$

Our Eq. (13) is similar to Maxwell–Schrödinger equations obtained in earlier works^33^, however Eq. (13) differs from previous works due to incorporating dissipation and dispersion of the surface-plasmon mode to the atomic medium’s evolution.

*Assumptions and feasibility in experiment* Now we present our assumptions to achieve spatiotemporal dynamics of the weak plasmonic field in the presence of polaritonic superradiant emission and next we give realistic parameters to test the feasibility of the scheme. Here, the atomic medium are coupled to a weak signal and orthogonally polarized strong driving fields, hence the atomic transition amplitude would be affect by these injected fields. Consequently, $c_{\imath ,\text {s}}$; $\imath \in \{1,\text {a},3\}$ represents the $| {\imath } \rangle$ atomic states affect by signal field and $c_{\imath ,\text {d}}$ describes its evolution for driving field. We achieve dynamical evolution of the weak field Eq. (13) and amplification by assuming modifications to atomic transition amplitudes as $c_{\imath }=c_{\imath ,\text {s}}+c_{\imath ,\text {d}}$, coherent term as $\tilde{\rho }_{31}=\tilde{\rho }_{31,\text {s}}+\tilde{\rho }_{31,\text {d}}$, Rabi frequency as $\Omega (\varvec{r},t)=\Omega _\text {s}(\varvec{r},t)+\Omega _\text {d}(\varvec{r},t)$. The weak signal plasmonic field then experience amplification and Eq. (13) describes its spatiotemporal dynamics.

In obtaining Eq. (13), we neglect the temporal evolution of the ground state $\dot{c}_{1,s}\ll 1$, assume copropagating electric signal and driving fields $\varvec{E}=\varvec{E}_\text {s}+\varvec{E}_\text {d}$, we consider the phase of the plasmonic field ($\varphi =\varvec{k}_\text {d}\cdot \varvec{r}-\omega t$) to be constant. Here, we test the feasibility of our scheme by employing the realistic parameters. For atomic medium $\omega _{21}=2.54~\text {eV}$ and $\omega _{31}\approx 2.04~\text {eV}$^76,77^. We set $I_\text {p}=30~\upmu \text {W}$ and propagation length as $L=200~\upmu \text {m}$. To describe NIMM layer we use Drude-Lorentz model with $\varepsilon _{\infty }=\mu _{\infty }=1.2$, $\omega _\text {e}=1.37\times 10^{16}~\text {s}^{-1}$, $\omega _\text {m}=10^{15}~\text {s}^{-1}$, $\gamma _\text {e}=2.37\times 10^{13}~\text {s}^{-1}$ and $\gamma _\text {m}=10^{12}~\text {s}^{-1}$^51^. Then for $\omega _{31}$ frequency transition $\text {Im}[\mathcal {A}(z,z_\text {at};k_\text {SPP})]\approx 3.2\times 10^{10}$ and $v_\text {SPP}\approx 2.61\times 10^{-2}\text {c}$. We employ Eqs. (7) and (8) commensurate with these realistic parameters to achieve the SSPP dynamics.

#### Weak plasmonic field amplification and stability analysis

In this section, we obtain sufficient conditions for weak polaritonic amplification within our hybrid plasmonic interface. Although this amplification is achieved for various system parameters, we assume specific modulation for coupling field intensities and detunings for efficient propagation of the signal plasmonic field and discuss the amplification stability. Now, we present the results for the case that a linearly polarized weak signal and orthogonally polarized strong driving fields are injected to our interaction interface.

We employ slowly varying amplitude approximation in our analysis^78^, assume $\gamma _1\approx 0$, and we take $\gamma _\text {SPP}$ to be a perturbation parameter due to the controllability of the virtual gain by external laser fields. Therefore, solving Eq. (13) commensurate with the Schrödinger equation for driving ($\Omega _\text {d}^{(1)}\approx \Omega _\text {d}^{(2)}:=\Omega _\text {d}$) and weak signal plasmonic fields and keeping the terms up to $\gamma _\text {SPP}$ and $\omega _\text {SPP}$, yields

15 $$\begin{aligned} \delta \omega _\text {SPP}\approx \tilde{\Delta }+\frac{|\Omega _\text {d}|^2}{\omega _\text {SPP}}\left[ 2+\text {i}\frac{\gamma _\text {SPP}}{\omega _\text {SPP}}\left( 1-\frac{|\Omega _\text {d}|}{\omega _\text {SPP}}\right) \right] . \end{aligned}$$

Note that all the terms within Eq. (15) are frequency shifts. The additional term, which depends on the driving field amplitude, would corresponds to Stark shift^79^ that limits the amplification efficiency in the experiment. This unwanted effect would also reduce the amplification efficiency in our system. First we note that leading term in Eq. (15) depends on driving field intensity that we refer as Stark shift^33^. The effect perturbs the excitation frequency of the SPP field due to spectral broadening, thus the SPP field frequency deviates from characteristic frequencies for which amplification can be achieved ($\omega _\text {SPP}\ne \omega _\text {ch}$). The Stark shift then destroys parametric resonance, suppresses the gain and consequently reduces the amplification efficiency.

In order to reduce this effect and efficient amplification, we suggest the driving field amplitude modifies within the interaction plane (*x*–*y*) as

16 $$\begin{aligned} \Omega _\text {d}(x,t)=\bar{\Omega }_\text {d}^{(1)}\text {e}^{\text {i}(\varvec{k}_\text {d}\cdot \varvec{r}-\omega _\text {d}t)}\hat{\epsilon }_{+}+\bar{\Omega }_\text {d}^{(2)}\text {e}^{-\text {i}(\varvec{k}_\text {d}\cdot \varvec{r}-\omega _\text {d}t)}\hat{\epsilon }_{-}, \end{aligned}$$

and its wavenumber modulates as $\varvec{k}_\text {d}\approx \varvec{k}_\text {SPP}+\delta \varvec{k}$ for $\delta \varvec{k}\ll \varvec{k}_\text {SPP}$. The resonant interaction between these weakly modulated plasmonic fields and directional SSPP preserves quantum coherence, reduce the unwanted Stark shift, and thereby provides efficient spectral component. In this case, Eq. (13) also changes to the Mathieu’s differential equation that supports the amplification of the weak signal field within characteristic dispersion curves^80^.

**Figure 4:**
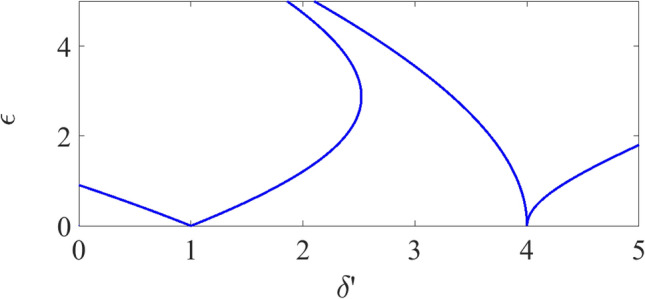
Stability diagram of the amplified SPP field: The presented curves in $\epsilon -\delta '$ plane denote the efficient amplification. Here, $\epsilon$ is the stiffness parameter that characterise the parametric amplification, and $\delta '$ is the re-normalized quantity that describe the parametric resonance (see Supplementary Information S8.1 for more information). Simulation parameters are $\beta =0.001$, $\omega _\text {d}=2\pi \times 20~\text {MHz}$, $m=1$, and we assume $\varvec{\nabla }\Omega _\text {s}=0$, $|\Omega _\text {d}|^2=10~\text {MHz}$, $\omega _\text {SPP}=\omega _{31}$ and $\gamma _\text {SPP}=0.004~\text {s}^{-1}$. Other parameters are the same as Fig. 3.

Specifically, we achieve the coherent amplification and investigate its stability within the atomic medium-NIMM interface in the case in the presence of directional plasmonic superradiant excitation Eq. (4) within our interaction interface. For our proposed realistic parameters, $\delta \varvec{k}_\text {d}\ll 1$ and by taking into account Eq. (16), we achieve

17 $$\begin{aligned} \tilde{\rho }_{11}-\tilde{\rho }_{33}\approx [1+\eta \cos (2\delta t)], \end{aligned}$$

$\eta$ the modulation parameter that depends on the plasmonic noise^30^. Now we define $\delta '$ as normalized detuning, and $\epsilon$ the normalized amplitude that depends on system parameters. We assume these quantities as our control parameters and investigate the amplification efficiency for this tunable system parameters. In this case, Eq. (13), which represents our simplified Maxwell–Schrödinger equation then reduces to Mathieu-like equation

18 $$\begin{aligned} \ddot{\Omega }_\text {s}+\delta '[1+\epsilon \cos (2\delta t)]\Omega _\text {s}=0, \end{aligned}$$

for $$\tilde{\Delta }\ll 1$$, and

19 $$\begin{aligned} \delta '=\mathcal {C}f(x,t)+v_\text {g}^{2}k_\text {SPP}^{2}/4, \end{aligned}$$

20 $$\begin{aligned} \epsilon =\frac{\mathcal {C}f(x,t)\eta }{\mathcal {C}f(x,t)+v_{g}^{2}k_\text {SPP}^{2}/4}. \end{aligned}$$

Equation (18) is quite similar to Mathieu’s equation that possess weak field amplification, and consequently this weak signal field would amplify in the presence of directional SSPP emission through parametric amplification. To achieve amplified plasmonic field without the need to population inversion, we solve Eq. (18) for weak plasmonic field modulation. Here we consider the initial plasmonic amplitude as $\Omega _\text {s}^{(0)}=0.5~\text {MHz}$ and introduce noise as $|\eta |=0.08\Omega _\text {s}^{(0)}$, and we finally assume $\dot{\Omega }_\text {s}(t=0)=0$^30^.

Our Mathieu equation Eq. (18) is achieved by applying the modulated polarized driving field and adjusting system parameters to Eq. (13). The parameters that describe the Mathieu equation, would consequently relate to the parameters describing Eq. (13) that depend on coupling lasers and waveguide parameters. Moreover, the parameters in Eq. (18) would also depend on weak signal field injection,that depends on plasmonic noise. This noise will affect the population difference due to Eq. (17) and consequently affects the Mathieu equation parameters. We express these parameters in terms of system parameters in Eqs. (19) and (20).

We then achieve the amplification based on two different mechanisms.

- Exploiting multiple-scale time variable techniques^80^. We perturbed the time as $t_{l}=\varepsilon ^{l}t$, $l\in \{0,1\}$ and we assume the asymptotic expansion to signal field Rabi frequency as $\Omega _\text {s}=\Omega _\text {s}^{(0)}+\mathcal {O}(\Omega _\text {s}^{(1)})$. The zeroth-order Ansatz $\Omega _\text {s}^{(0)}\sim \exp \{\delta 't\}$ amplifies for specific oscillation frequency $\omega _0$, which is different from oscillation frequency of quantum SPP mode $\omega _\text {o}$, if $\delta ':=\omega _\text {0}^2$ and $\delta \approx \omega _\text {0}/m$ with $m=1,2,3,\ldots$. Here, the nonlinear processes generate frequency combs ($\delta \omega _\text {comb}\approx 10~\text {MHz}$^5^) that leakage the SPP energy to other spectral component, and reduce amplification efficiency. Consequently, the frequency grid should be small compared to this frequency comb spacing.
- Using the gain modulation. To obtain the gain threshold we set $\delta '=\delta '^{(0)}+\varepsilon \delta '^{(1)}+\mathcal {O}(\delta '^{(2)})$, $\Omega _\text {s}=\Omega _\text {s}^{(0)}+\varepsilon \Omega _\text {s}^{(1)}+\mathcal {O}(\Omega _\text {s}^{(2)})$, consider the zeroth order solution as $\Omega _\text {s}^{(0)}\sim A\text {e}^{\text {i}t_0}$, and first order perturbation as $\Omega _\text {s}^{(1)}:=A(\delta ')\exp \{\delta t\}$, for $A(\delta '):=\text {Re}[A(\delta ')]+\text {i}\text {Im}[A(\delta ')]$ the amplitude of the superradiant mode under weak perturbation limit. Plugging into Eq. (18) we obtain the gain threshold for amplification of the weak SPP wave as
  21 $$\begin{aligned} 2\gamma _1\approx \sqrt{1-4\delta '^{(1)^2}}. \end{aligned}$$
  The plasmonic system provides gain for the detuning frequency characterized by Eq. (21). As high frequency deviation produces gain at least for two specific frequencies, which reduces the amplification efficiency, the frequency deviation should be narrow to produce gain only for $\omega _0$.

**Figure 5:**
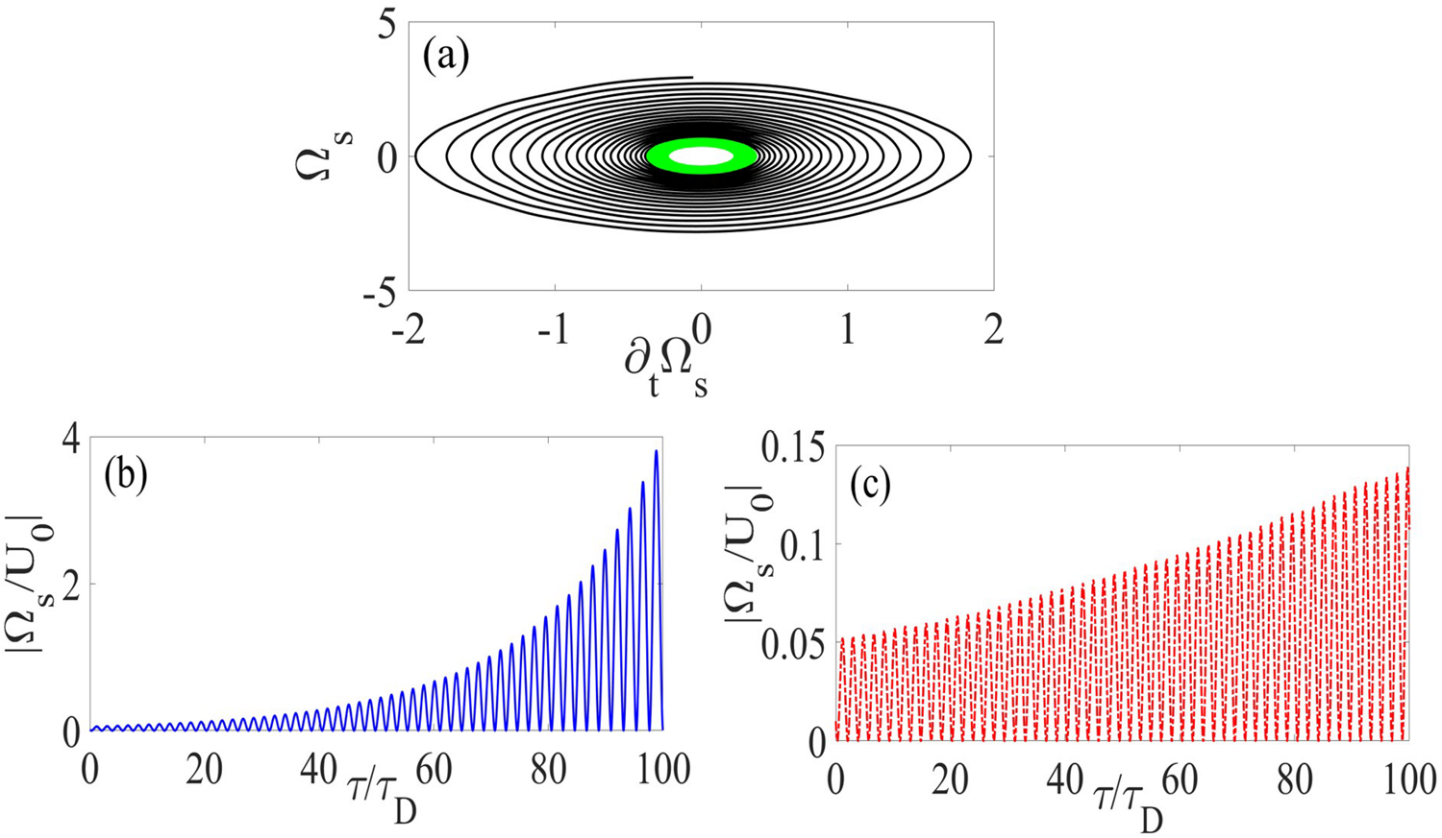
Dynamics of the SSPP: The figure represents the superradiant emission with efficient amplification condition Eq. (22) (blue solid line) and without considering Eq. (22). The parameters for these plots are $|\Omega _\text {d}^{(0)}|=10~\text {MHz}$ (for red line), $\delta '^{(0)}=0$, $\delta '^{(1)}=\omega _0$. Other parameters are the same as Figs. 3 and 5.

The stable amplification for sufficiently large $\delta '-\epsilon$ is induced to this chaotic plasmonic interface through resonant interaction between weak plasmonic field and SSPP emission^81^. In this case, Eq. (18) is a homogeneous equation that yields stable amplification of the weak plasmonic field within certain spectral region represented in Fig. 4. Specifically, our simulation demonstrates that the efficient SPP amplification with $\epsilon \ne 0$ can be achieved for $1<\delta '<4$. We extend our model to the spatially dependent amplification of the SSPP by considering ($\varvec{v}_\text {SPP}\cdot \varvec{\nabla }$) as a correction term and treating $\beta$ as a perturbation term. The weak plasmonic field is modulated as

$$\begin{aligned} \Omega _\text {s}:=\Omega _{s}^{(0)}[1+p(x)]\exp \{\delta t+\text {i}\mathcal {K}x\}, \end{aligned}$$

with $\mathcal {K}:=|\varvec{k}_\text {SPP}|$. Now we substitute this equation into Eq. (10) and and employ linear stability analysis^82^. In this case, the nonlinearity would be reduced and this SPP wave amplifies within the spectral stability diagram represented in Fig. 4 if the system parameters are modulated as

22 $$\begin{aligned} \frac{4\Omega _\text {d}^{2}}{\omega _\text {SPP}+\text {i}\gamma _\text {SPP}}+\text {i}\mathcal {K}|\varvec{v}_\text {SPP}|\approx 0, \end{aligned}$$

and we assume $\gamma _\text {SPP}=0.025~\text {cm}^{-1}$. The amplification pattern corresponds to this propagated plasmonic mode is represented in Fig. 5. The robustness of amplification depend on low SPP field propagation and robust directional SSPP launching. Due to loss-compensation scheme, we expect the stable propagation of SPP field and hence robust amplification of SPP field is produced only for robust SSPP, which is achieved as waveguide decay rate ($\Gamma _\text {W}$) exceed the free-space one ($\Gamma _\text {F}$)^83^. For our hybrid waveguide the decay rate is controllable, $\Gamma _\text {W}=5\times 10^{-3}\gamma _\text {F}$, and a specific case of $m=N_\text {a}$ is also achievable. For this much photon collections and atomic excitation, robust SSPP are expected with error scale up to $\varepsilon _\text {er}\approx 0.12$. Therefore, the SSPP radiation and SPP lasing operation for both $m\ll N_\text {a}$ and $m\approx N_\text {a}$ limits are achievable with fairly low error scale, which demonstrate the robustness of our scheme.

### Detection of amplified plasmonic field

This amplified plasmonic wave can be detected in an experiment. We underpin our method for measuring the amplified SPP field, based on the far-field pattern of the propagated plasmonic waves. We define this far-field radiation $\varvec{E}_\text {super}$ in terms of Poynting vector as $\varvec{S}=(1/2)\text {n}_\text {eff}\varepsilon _{0}|\varvec{E}(\varvec{r},t)|^{2}\varvec{e}_{\parallel }$, for $\text {n}_\text {eff}$ the effective refractive index of the interface and $\varvec{e}_\parallel$ the unit vector of the superadiant field along the interface. This electric field is confined to the interface with an effective thickness $z=z_\text {at}$, and we evaluate this plasmonic field for our hybrid interface in Supplementary Information S11. The intensity profile of this signal plasmonic wave thereby depends on the interference pattern of this far-field emission, which is constructive for azimuth angle $\phi$ and polar angle $\theta$ satisfying

23 $$\begin{aligned} \mathcal {K}\sin \theta (\cos \phi x+\sin \phi y)=2m\pi . \end{aligned}$$

For measuring the amplified plasmonic wave intensity, we suggest a detection system that is placed in a spatial coordinate obtained by solving Eqs. (3) and (23) (we represent the detailed derivation of the Eq. (23) in the Supplementary Information S11).

**Figure 6:**
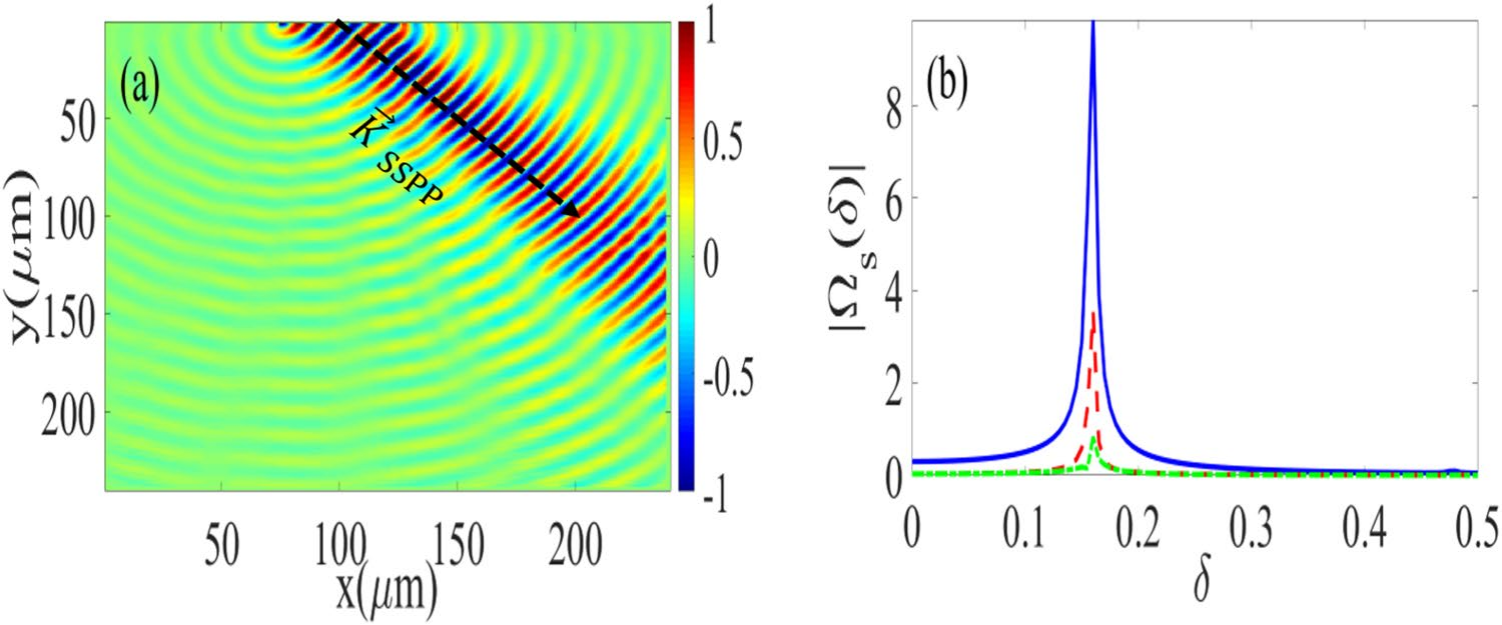
Panel (**a**) represents a field pattern for a $200\times 200~\upmu \text {m}^2$ plasmonic waveguide obtained by finite difference time domain method. Parameters are: $I_\text {s}=30~\upmu \text {W}$, $(2\text {n}_{p}+1)\sim 19$, $I_\text {d}=10~\text {m}\text {W}$. Other parameters are the same as Fig. 3. Panel (**b**) is the spectral evolution of the weak signal field pulse, and SPP lasing operation. The parameters are: $\gamma _\text {SPP}=0.01~\text {cm}^{-1}$ for blue curve, $\gamma _\text {SPP}=0.03~\text {cm}^{-1}$ for dashed red curve and $\gamma _\text {SPP}=0.1~\text {cm}^{-1}$ for green dotted dashed curve. Other parameters are the same as Fig. 4.

We detect this wave for a certain deviations in detuning, amplitude and frequencies. This effect is detectable for deviation in frequency detunings that introduce negligible atomic absorption and provide loss-compensation. Also, for signal amplitudes with suppressed intensity dependent nonlinear effects such as Kerr nonlinearity and higher-order dispersion, an amplification with coherent spectral properties is expected. Finally, only those frequency deviations can be efficiently scattered and detected via our plasmonic configuration that are resonant with our characteristic Bragg frequencies. Now we test the directionality and stability of this signal field, by assuming a $200\times 200~\upmu \text {m}^2$ of our proposed device, subjected to a optical pump with intensity $I_\text {s}=30~\upmu \text {W}$, and a train of $(2\text {n}_{p}+1)\pi$ pulses similar to Ref.^16^ that would excite superradiant pulse at $x\approx 75\;\upmu \text {m}$. We suppose signal and driving fields are injected at $(x_0,y_0)=(75 \;\upmu {\text {m}},0)$. Then we simulate the field pattern using finite difference time method^84^. Our simulation represents that the weak signal plasmonic field stably propagates and detects in the direction characterized by SSPP propagation, as we have clearly shown in Fig. 6a.

## Discussion

We begin this section by summarizing our quantitative and qualitative approaches towards weak plasmonic field amplification without need to population inversion at the interface between atomic medium and NIMM layer. Quantitative description of this amplification process is achieved through three main equations. First, we develop Eq. (7) to describe directional plasmonic superradiant emission. Next, we achieve and employ Maxwell–Schrödinger equation, Eq. (13) to elucidate the dynamics of the weak plasmonic field within our dissipative interface. Finally, we achieve Mathieu-like system, Eq. (18), to establish stable amplification of SPP waves without the need for population inversion.

Our methodological approach for coherent and inversionless amplification of the weak SPP field is based on three main concepts, namely, (i) launching directional superradiant emission, (ii) ultra low-loss propagation of weak SPP field, and (iii) parametric resonance and field amplification. Directional launching of superradiant emission is a valid concept, which is experimentally verified for a chain of one-dimensional atomic medium^85^. Here, we assume $\text {Pr}^{3+}$ impurities within a transparent crystal that acts as a one-dimensional chain, and hence our methodology to exciting directional SSPP is valid due to experimental feasibility. Next, our amplification scheme is also based on ultra-low loss propagation of the weak SPP field, which is a valid concept and the experimental/theoretical feasibility has been extensively discussed recently^67^. Finally, we elucidate field amplification through parametric resonance, which is a valid methodology for inducing amplification in dynamical systems such as micro-electro-mechanical systems^86^ and superconducting cavities^87^. Our methodology thereby is based on concepts that are experimentally/theoretically valid.

Qualitatively, to achieve efficient amplification, we reduce the effect of inhomogeneous broadening related to the atomic medium by proposing the persistent spectral hole-burning^88^, that can be achieved by employing a pump field with high-optical density and an auxiliary re-pump field and for scanning spectral hole, we suggest intensity-reduced single-mode laser operation with tunable cavity. To achieve ultra-low Ohmic loss for optical frequencies, we suggest nano-fishnet structures commensurate with virtual gain induction. Coherent amplification of weak plasmonic field without the need for population inversion can then be tested by employing achievable technologies such as cold atoms, ultra-low loss metamaterials and finely tuned laser fields.

Our suggested amplification scheme is based on directional superradiant emission, that can be achieved for evanescent electromagnetic fields within different platforms such as tapered nanofibers^89,90^ or nanophotnic waveguides^91^. Consequently, superradiant emission and stable amplification of the weak SPP field should also achieve by considering an atomic chain doped on a plasmonic nanowire. The loss related to this nanowire scheme, quantum dephasing and providing quantum gain through Mathieu equation within this hybrid nanowire-atomic medium interface need further consideration and can be considered as a future work. In this paper, we exploit achievable technologies such as cold atoms and ultra-low Ohmic loss metamaterials to achieve coherent amplification of the SPP field with negligible dephasing even for a very low input plasmonic field.

As we establish in “Results”, the weak SPP wave amplifies within the atomic medium-NIMM layer interface through resonant coupling between this plasmonic mode and directional superradiant SPP. Our method takes advantage of the constructive interference between two contra-propagating plasmonic modes to suppress the Ohmic loss, and we achieve stability by coupling the atomic ensemble dynamics of injected signal plasmonic waves. This quantum-plasmonic configuration, thereby serves as an efficient plasmonic transistor^92^. The operation of this device is based on the generation of controllable output plasmonic fields for a weak input SPP signal. Using the realistic parameters for the atomic medium and for the NIMM layer, and by controlling the external laser-field intensities, the Ohmic loss and consequently the output power of the SPP field can be coherently controlled. Therefore, our scheme acts as a field-effect plasmonic transistor with a controllable fast-switch that operates in the optical frequency range.

Our proposed apparatus also serves as a surface plasmon laser. We explain the lasing operation is based on three effects: (i) this scheme preserves coherence due to quantum decoherence suppression and exploiting coherent loss-compensation mechanism, (ii) we achieve a strong directionality for the amplified SPP wave by resonant interaction between directional superradiant SPP emission and weak plasmonic field, and (iii) our amplification scheme prevents the generation of amplified spontaneous emission of the plasmonic wave^21^ due to weak-field seeding and the spectral width of this intense emission is much narrower compared to other schemes due to limitations induced by the collective oscillation of the atomic medium. This lasing operation depends on effective of the SPP mode, and spectral properties and amplification coefficient for a stable frequency is dramatically enhanced by suppressing the loss related to guided SPP mode. Plasmonic loss is another practical imperfection, and lasing operation of SPP is achieved for SPP loss less than $\alpha <0.03~\text {cm}^{-1}$, as it is clearly shown in Fig. 6b. We achieve this spectrum by employing a Fourier transform to numerical solution of Eq. (18). To suppress this unwanted loss we suggest virtual gain technique, which is extensively discussed in Ref.^67^. This coherent amplification without need to population and high-input power of SPP fields thus open prospects for applications in wide research areas from biology and sensing to interconnects^18^.

## Conclusion

In summary, we devise a quantum-plasmonic waveguide, that exploits superradiant emission of radiation to produce inversion-less SPP lasing and coherent intense SPP waves even for weak input plasmonic pulses. Our scheme incorporates an experimentally feasible source-waveguide-detection triplet and the waveguide (as a sub-element of our apparatus) is a hybrid structure comprises atomic medium doped in a transparent dielectric situated above a NIMM layer. We establish SSPP dynamics based on non-Markovian spontaneous emission of atomic ensemble, which is achieved by coupling quantum plasmonic mode to collective atomic oscillation.

Our framework for spatiotemporal dynamics of the weak plasmonic field within the interaction interface is based on Fourier component evolution, yielding coupled Maxwell–Schödinger like equation. We employ a coherent loss-compensation scheme and establish a resonant interaction between SSPP mode and signal plasmonic field to suppress the quantum decoherence of the amplified directional SPP wave. The quantum gain for this weak seeded plasmonic field is produced by introducing parametric resonance between SSPP and modulated stable plasmonic field. We theoretically evaluate and numerically demonstrate that this weak SPP field stably propagates within the direction characterized by launched directional SSPP. Our amplification scheme is efficient and robust against the photon loss and is only achieved for specific errors or deviations in detuning, amplitude and frequencies, specifically for wide range of collective excitation from $m\ll N_{a}$ to $m=N_\text {a}$. We have justified that the output SPP field is spectrally efficient with suppressed amplified spontaneous emission even under these practical imperfections. Consequently, our inversion less concept is analyzed for experimentally feasible configuration from source to detection, which introduces a novel concept for coherent amplification of SPP waves and should act as plasmonic field-effect transistor and surface plasmon lasers.

## Methods

In this work, dynamics of SSPP in the interface between the atomic medium and nano-fishnet metamaterial layer and stable amplification of the weak plasmonic wave without need to population inversion are obtained by employing the multiple scaled time and asymptotic expansion to Mathieu equation and Fourier optics of surface polaritonic wave. Consequently, first, we describe our perturbation technique by elucidating asymptotic expansion commensurate with multiple scale variables in “Multiple-scale variable and asymptotic expansion” and next, we briefly discuss the Fourier optics of SPP waves in “Fourier optics of surface plasmons”. Finally, we use these mathematical techniques and employ the concepts presented in background to describe the amplification of the weak surface polaritonic field in the presence of directional of SSPP radiation.

### Multiple-scale variable and asymptotic expansion

Our methods for solving the Mathieu/Hill equations within this hybrid plasmonic waveguide is based on multiple scale time variable commensurate with the asymptotic expansions^80^.

Weak signal field Rabi frequency ($\Omega _\text {s}(x,t)$) possesses temporal and spatial dynamics through propagating along hybrid interface. We achieve the evolution of this plasmonic field using perturbation methods. Our perturbation method is based on asymptotic expansions with multiple scale time (*t*) and position (*x*) and we truncate the perturbation up to first order. Consequently, we consider $x_1=\varepsilon x$ and $t_l=\varepsilon t$ for $\varepsilon :=|\Omega _\text {s}/\Omega _\text {d}|$ the order of perturbation. Our mathematical treatment, such as Maxwell–Schrödinger equation is then achieve through linearization of the signal field Rabi frequency dynamics. We left the mathematical details towards asymptotic expansion, order of perturbation and multiple scale variable to the Supplementary Information S9.2. Here, we employ this concept to solve the resultant Mathieu differential equation perturbatively to achieve the sufficient condition for plasmonic field amplification without the need to population inversion.

### Fourier optics of surface plasmons

Our method for calculating quantized electric field is based on Green function method^93,94^ that is a Dyadic tensor. This dyadic Green function for surface polaritonic waves can be obtained in two different mechanism, i.e. (i) real frequency ($\omega$) and complex wavenumber *k*, and (ii) complex frequency and real wavenumber^72^. For a characterized plasmonic Green tensor within a dissipative hybrid interface, the quantized electric field is then related to the Green tensor with Supplementary Eq. (S35) (see Supplementary Information S9.2 of the supplementary material for more mathematical details). We assume relative position $\delta \varvec{r}=\varvec{r}-\varvec{r}'$ and relative time $\delta t=t-t'$ to express this Green function in a Fourier space as

24 $$\begin{aligned} \varvec{\mathcal {A}}(\varvec{r},\varvec{r}';\omega )=\int \frac{\text {d}^2\varvec{q}}{(2\pi )^2}\int \frac{\text {d}\tilde{\omega }}{2\pi }\varvec{g}(\varvec{q},z,z';\tilde{\omega })\text {e}^{\text {i}[\varvec{q}\cdot \delta \varvec{r}-\tilde{\omega }\delta t]}. \end{aligned}$$

We interpret (24) as the general dyadic Green tensor for a dissipative interface and we describe the propagation properties of the SSPP and dynamical evolution of the weak plasmonic field using (24).

Aforementioned explanation is valid for a propagating surface-plasmonic wave in our hybrid interface. Consequently, our quantum SPP should also be described using complex wavenumber or complex frequency representations in the Fourier space. This representation would depend on the zeros of the propagation constant. For a NIMM layer, propagation constant depend on the frequency dependent electric permittivity $\varepsilon _\text {N}$ and magnetic permeability $\mu _\text {N}$ that is characterized through macroscopic Drude-Lorentz as

25 $$\begin{aligned} \varepsilon _\text {N}=&\varepsilon _{\infty }-\frac{\omega _\text {e}^2}{\omega _l(\omega _l+\text {i}\gamma _\text {e})}, \end{aligned}$$

26 $$\begin{aligned} \mu _\text {N}=&\mu _{\infty }-\frac{\omega _\text {m}^2}{\omega _l(\omega _l+\text {i}\gamma _\text {m})}, \end{aligned}$$

for $\varepsilon _{\infty }$ and $\mu _{\infty }$ the background constant for the permittivity and permeability, respectively. The other constants are $\omega _l$ the perturbation frequency, $\omega _\text {e}$ ($\omega _\text {m}$) are the electric and magnetic plasma frequencies, and $\gamma _\text {e}$ ($\gamma _\text {m}$) are the corresponding decay rates. Now we present this explanation in mathematical details. To this aim, first, we note that the SPP field dispersion in the interface between a dielectric and a NIMM layer is

27 $$\begin{aligned} q(\omega _l)=\frac{\omega _l}{c}\sqrt{\frac{\varepsilon _\text {N}\varepsilon _0(\varepsilon _\text {N}\mu _0-\varepsilon _0\mu _\text {N})}{\varepsilon _\text {N}^2-\varepsilon _0^2}}. \end{aligned}$$

The roots correspond to this propagation constant (i.e. $q(\omega _l)=0$) in our interactive interface is achieved in two alternative mechanisms, (i) considering $\omega _l$ as a real parameter to find a complex root for wavenumber, or (ii) introducing a real value $\varvec{q}$ to find the complex root of perturbation frequency. Consequently, the Green tensor related to this SPP dispersion can also be evaluated in terms of these considerations.

Next, we employ residues theorem^95^ to calculate the plasmonic Green function for roots in complex $\tilde{\omega }$-real $|\varvec{q}|$ Fourier space. We assume $\omega _\text {SPP}\approx \omega _{31}$ as the frequency excitation of our plasmonic mode. Now, the plasmonic Green tensor can be represented for complex $\varvec{q}$ or complex $\tilde{\omega }$. The plasmonic Green tensor ($\varvec{\mathcal {A}_\text {SPP}(\varvec{q},z,z')}$) corresponds to this complex SPP frequency excitation $\omega _\text {SPP}$ and $-\omega _\text {SPP}^{*}$ becomes

28 $$\begin{aligned} \varvec{g}(\varvec{q},z,z';\tilde{\omega })=\frac{\mathcal {A}_\text {SPP}(\varvec{q},z,z')}{\tilde{\omega }-\omega _\text {SPP}}+\frac{\mathcal {A}_{-\text {SPP}}(\varvec{q},z,z')}{\tilde{\omega }+\omega _\text {SPP}^{*}}, \end{aligned}$$

for a real *q* space, whose components are characterized by $q_x,q_y$. Alternatively, by considering real $\omega _{l}$ and complex wavenumber $\varvec{q}$, this Green tensor can be achieved in terms of characteristic complex SPP wavenumber $\varvec{k}_\text {SPP}$ and $-\varvec{k}_\text {SPP}^{*}$ as

29 $$\begin{aligned} \varvec{g}(\varvec{q},z,z';\tilde{\omega })=\frac{\mathcal {A}_{q_{x}}(q_{z},z,z';\tilde{\omega })}{q_{x}-k_\text {SPP}}+\frac{\mathcal {A}_{-q_{x}^{*}}(q_{z},z,z';\tilde{\omega })}{q_{x}+k_\text {SPP}^{*}}. \end{aligned}$$

In this work, we develop our method and calculate the Green tensor of the atomic medium-NIMM layer interface based on surface plasmon Fourier optics^72^.

## Supplementary information

Supplementary Information.
