# Supplementary material for: Coherent amplification and inversion less lasing of surface plasmon polaritons in a negative index metamaterial with a resonant atomic medium
Source: Sci Rep. 2021 Feb 10;11:3450. doi: 10.1038/s41598-021-82909-7 (PMC7876136; doi:10.1038/s41598-021-82909-7)
Supplement: Supplementary file 1 — Supplementary Information. [file 41598_2021_82909_MOESM1_ESM.pdf]

Supplementary material: Coherent amplification and inversion less lasing of surface plasmon polaritons in a negative index metamaterial with a resonant atomic medium

Saeid Asgarnezhad-Zorgabad[1,*]
[1]Department of physics, Sharif university of technology, 11165-9161, Tehran, Iran
*sasgarnezhad93@gmail.com

Abstract

In this supplementary material, we provide necessary mathematical steps towards weak SPP field amplification. Consequently, this section represents the quantitative description of the weak SPP lasing. To achieve this coherent amplification, we derive and employ three equations: (i) directional superradiant emission, Eq. (7), (ii) coupled Maxwell-Schrödinger equation, Eq. (13), and (iii) Mathieu-like equation, Eq. (18). In this supplementary materials, first, we elucidate the mathematical aspects of Mathieu's differential equations, multiple scale variables and asymptotic expansion as a basic of our mathematical description in § 8. Next, we elucidate the dynamical evolution of superradiant plasmonic field in the limiting case of $\Omega_{\rm s}=\Omega_{\rm d}=0$ in § 9. Finally, we explore mathematical steps toward weak plasmonic field and stability analysis weak SPP in § 10. Specifically, we employ Schrödinger approach and derive Eq. (7) of the main text. We also use Fourier optics of SPP waves to achieve the dynamics of weak plasmonic fields and hence derive Eq. (13) of the main text.

8 Mathematical methods towards weak SPP field amplification

In this section we briefly review the basic mathematical steps towards weak SPP field amplification. To this aim, we employ two mathematical concepts, i.e., (i) Mathieu's equations, and (ii) multiple scale variables and asymptotic expansion. Consequently, first in § 8.1 we review the main mathematical consequences of the Mathieu equation and in § 8.2 we discuss the multiple scale variable and asymptotic expansion methods.

8.1 Mathieu, Hill Equations: Parametric resonances and stability consideration

Mathieu equation describes the dynamical evolution of the system with nonlinear vibrations through a linear second order ordinary differential equation

$$\frac{\mathrm{d}^2x}{\mathrm{d}t^2}+(\delta+\epsilon\cos t)x=0, \tag{S1}$$

for δ and ϵ the control parameters of the system. This equation describes the simple harmonic motion with a resonance frequency $\delta=\omega_0^2$ in the limiting case $\epsilon\mapsto 0$. However, for $\epsilon\neq 0$, the interplay between δ and ϵ yield stable vibration of variable $x(t)$. $x(t)$ would stably vibrate if the motion can stay *bounded*, and $x(t)$ is unstable for unbound motion of the physical quantity. We refer corresponding δ-ϵ diagrams characterising the stability of the vibrations as stability chart or Strutt-Ince diagram[S1].

In our system, the weak plasmonic field irradiates the waveguide as modulated periodic plane wave, and the stability of the Mathieu equation can be obtained through Floquet theory. To achieve the origin of the stability diagram, we begin with the most general case of Mathieu equation, known

as Hill's equation

$$\frac{\mathrm{d}^2x}{\mathrm{d}t^2}+f(t)x=0, \quad f(t+T)=f(t), \tag{S2}$$

and assume $x_1 = x$, $x_2 = \dot{x}$. The Hill's equation then becomes

$$\frac{\mathrm{d}}{\mathrm{d}t}\begin{bmatrix}x_1\\x_2\end{bmatrix}=\begin{bmatrix}0 & 1\\-f(t) & 0\end{bmatrix}\begin{bmatrix}x_1\\x_2\end{bmatrix}, \tag{S3}$$

We take the solution of the Eq. (S3) at $t = T$ as

$$C=\begin{bmatrix}x_{11}(T) & x_{21}(T)\\x_{12}(T) & x_{22}(T)\end{bmatrix}, \tag{S4}$$

the eigenvalue of this matrix λ

$$\lambda^2-\mathrm{tr}(C)\lambda+\det(C)=0, \tag{S5}$$

would characterize the region with unstable vibrations for which a physical quantity x can be exponentially growth during the time interval. In this work, we assume weak SPP field as a periodic intial condition and consider the unbounded solution of the probe field Rabi frequency for which this plasmonic field can amplify. Consequently, we achieve the field amplification for the central frequencies close to the SSPP frequency $\omega_\mathrm{s} = \omega_\mathrm{SSPP}$.

8.2 Multiple scale variable and asymptotic expansion

In this section we describe order of perturbation, multiple scale variable and asymptotic expansion. To define the order of perturbation, let us assume $\varphi_n : \mathbb{R}\setminus 0 \to \mathbb{R}$ as a sequence function for $x \to 0$ if for any $n \in \{1, 2, 3, \ldots\}$ there is a gauge function φ_n satisfying

$$\varphi_{n+1}=o(\varphi_n). \tag{S6}$$

Here, we write $f(\epsilon) = o[g(\epsilon)]$ as $\epsilon \to 0$ if for any positive number δ, independent of ϵ, there exist ϵ_0 such that $|f(\epsilon)| \leq \delta|g(\epsilon)|$ for $|\epsilon| \leq |\epsilon_0|$[S2]. Then asymptotic expansion of any arbitrary function $f(x)$ and characterized $N \in \mathbb{N}$ in terms of these sequence functions is defined as a serious

$$f(x)\sim\sum_{n=0}^{N}\epsilon^n\varphi_n, \tag{S7}$$

only if

$$f(x)-\sum_{n=0}^{N}\epsilon^n\varphi_n=o(\varphi_N). \tag{S8}$$

We can rewrite (S8) as

$$f(x)-\sum_{n=0}^{N-1}\epsilon^n\varphi_n=\mathcal{O}(\varphi_N). \tag{S9}$$

Similar to previous definition, we write $f(\epsilon) = \mathcal{O}[g(\epsilon)]$ as $\epsilon \to 0$ if for any positive number A, independent of ϵ, there exist $\epsilon_0 > 0$ such that $|f(\epsilon)| \leq A|g(\epsilon)|$ for $|\epsilon| \leq |\epsilon_0|$[S2].

9 Dynamical evolution Superradiant plasmonic field

In this section, we give a detailed explanation of exciting and launching directional SSPP emission, which is proportional to the excited $|3\rangle$ state within the interaction interface when the signal and driving field is switched off (i.e. $\Omega_s = 0$ and $\Omega_d = 0$). Our SPP mode with a wavenumber $\boldsymbol{k}_{\rm spp}$ propagates as superradiant emission through a collective excitation process if this single surface-plasmon field is uniformly absorbed by an ensemble of N_a atomic medium through time-Dicke state

$$|\psi_{\rm SPP}\rangle = \frac{1}{\sqrt{N_a}} \sum_{l=1}^{N_a} \exp\left[i\boldsymbol{k}_{\rm SPP} \cdot \boldsymbol{r}_l\right] |3_i\rangle \otimes_{j\neq l} |1_j\rangle \,. \tag{S10}$$

The spontaneous emission from this prepared atomic ensemble creates a SPP field with wavevector $\boldsymbol{k}$, and the energy $\hbar(\omega_3 - \omega_1)$. Similar to approach presented in[S3], this emission is superradiant and directional if $\boldsymbol{k} = \boldsymbol{k}_{\rm SPP}$ and $\omega_{\rm SPP} \approx \omega_{31}$. To satisfy these requirements, we suggest an optical pump and two contra-propagated couple laser fields as represented in Fig. 1 of the main text.

In our scheme, the optical pump is employed to induce coherence and hence provide collective excitation through $|2\rangle \leftrightarrow |1\rangle$ atomic transition and we employ $(2n_p + 1)$-π pulse to establish directionality. These pulse trains are resonant with $|2\rangle \leftrightarrow |3\rangle$ transition and their wavenumber $\boldsymbol{k}_{C\imath}$; $\imath \in \{1, 2\}$ provides a unidirectional superradiant SPP wavenumber with[S4]

$$\boldsymbol{k}_{\rm SPP} = (n_p + 1)\boldsymbol{k}_{C2} - n_p \boldsymbol{k}_{C1}. \tag{S11}$$

As this SPP field coupled with the atomic state $|3\rangle$, the spectral component of the temporal atomic evolution then describes the superradiant surface-plasmonic dynamics.

Here we provide necessary steps toward SSPP dynamics. This evolution is the Fourier spectrum of the c_3 atomic state in the case that both signal and driving fields are switched off. We obtain the temporal evolution by exploiting the Schrödinger equation approach. To this aim, first, we characterize the quantized current density $\hat{\boldsymbol{j}}$, quantized electric field $\hat{\boldsymbol{E}}$ and employ green function approach to evaluate the Hamiltonian of the system. Then we employ this Hamiltonian to achieve the dynamics of the excited atomic state.

9.1 Interaction Hamiltonian of the system

Next, we achieve the interaction Hamiltonian of this plasmonic scheme in three steps: (i) employ canonical quantization method, by introducing bosonic creation/annihilation operators to achieve the quantized current density within quantum emitter-NIMM layer interface, (ii) use this current to quantized the electric field component of SPP mode, and (iii) exploit a dipole approximation and express the Hamiltonian of the system in terms of our bosonic operators and atomic dipole moment.

We evaluate quantized current density $\hat{\boldsymbol{j}}(\boldsymbol{r}, \omega)$ in the interface between a dielectric and our NIMM layer interface by considering

$$\mu_N(\boldsymbol{r}, \omega) := [\kappa_N(\boldsymbol{r}, \omega)]^{-1}, \tag{S12}$$

and defining

$$\alpha(\boldsymbol{r}, \omega) = \left\{\frac{\hbar\varepsilon_0}{\pi} \mathrm{Im}[\varepsilon_N(\boldsymbol{r}, \omega)]\right\}^{1/2}, \tag{S13}$$

$$\beta(\boldsymbol{r}, \omega) = \left\{-\frac{\hbar}{\pi\mu_0} \mathrm{Im}[\kappa_N(\boldsymbol{r}, \omega)]\right\}^{1/2}, \tag{S14}$$

as[S5, S6]

$$\hat{\boldsymbol{j}}(\boldsymbol{r},\omega) = -\,2\pi\mathrm{i}\omega\alpha(\boldsymbol{r},\omega)\hat{\boldsymbol{C}}_{\mathrm{e}}(\boldsymbol{r},\omega) + 2\pi\boldsymbol{\nabla}\times\left\{\beta(\boldsymbol{r},\omega)\hat{\boldsymbol{C}}_{\mathrm{m}}(\boldsymbol{r},\omega)\right\}. \tag{S15}$$

Here we assume $\hat{\boldsymbol{C}}_{\jmath}(\boldsymbol{r},\omega)$ $(\hat{\boldsymbol{C}}_{\jmath}^{\dagger}(\boldsymbol{r},\omega))$; $\jmath\in\{\mathrm{e},\mathrm{m}\}$ as annihilation (creation) operators associated with the electrical (e) and magnetic (m) response of the medium, whose components are described by usual bosonic commutation relation

$$\left[\hat{C}_{\jmath i}(\boldsymbol{r},\omega),\hat{C}_{\jmath' j}(\boldsymbol{r}',\omega')\right] = 0, \tag{S16}$$

$$\left[\hat{C}_{\jmath i}(\boldsymbol{r},\omega),\hat{C}^{\dagger}_{\jmath' j}(\boldsymbol{r}',\omega')\right] = \delta_{ij}\delta_{\jmath\jmath'}\delta(\omega-\omega')\delta(\boldsymbol{r}-\boldsymbol{r}'). \tag{S17}$$

We calculate the quantized current density for our specific NIMM layer by plugging Eqs. (S39) and (S40) into Eqs. (S13) and (S14).

Next, we employ quantized current density characterized by Eq. (S15) to evaluate the quantized electric field operator within interaction interface. This electric field is related to the Dyadic green function at the interface $\mathcal{A}(\boldsymbol{r},\boldsymbol{r}';\omega)$. In the limiting case of dielectric-metamaterial interface, this green function can be calculated similar to Ref.[S7]. To this aim, first we define

$$\int_{\boldsymbol{r},\tilde{\omega}} := \mathrm{i}\frac{\mu_0}{2\pi}\int \mathrm{d}^3\boldsymbol{r}\int_0^{\infty}\mathrm{d}\tilde{\omega}, \tag{S18}$$

and then express the quantized electric field in terms of system parameters as

$$\boldsymbol{E}(\boldsymbol{r},t) = \int_{\boldsymbol{r}',\tilde{\omega}} \hbar\tilde{\omega}\left[\boldsymbol{\mathcal{A}}(\boldsymbol{r},\boldsymbol{r}';\tilde{\omega})\cdot\hat{\boldsymbol{j}}(\boldsymbol{r}',\tilde{\omega})\mathrm{e}^{\mathrm{i}\tilde{\omega}t} + \mathrm{h.c}\right]. \tag{S19}$$

We employ this quantized electric field to describe the interaction Hamiltonian for the interface between the NIMM layer and the atomic medium interface.

Finally we employ the quantized electric field, commensurate with bosonic annihilation/creation operators to evaluate the total Hamiltonian of the system. To this aim, we define the atomic energy levels by

$$E_j = \hbar\omega_j, \quad j\in\{1,2,3\} \tag{S20}$$

for each atomic state $|j\rangle$. We assume the dipole moment of the $|3\rangle \leftrightarrow |1\rangle$ as $\boldsymbol{d}_l$ and also introduce the Pauli matrices correspond to this atomic medium as

$$\sigma_l^z = |3_l\rangle\langle 3_l| - |1_l\rangle\langle 1_l|, \tag{S21}$$

$$\sigma_l^x = |3_l\rangle\langle 1_l| + |1_l\rangle\langle 3_l|. \tag{S22}$$

The Hamiltonian of this plasmonic scheme then becomes

$$H = \sum_{l=1}^{N_{\mathrm{a}}}\left[\frac{\hbar\omega}{2}\sigma_l^z - \sigma_l^x\boldsymbol{d}_l\cdot\boldsymbol{E}_l(\boldsymbol{r}_l)\right] + \sum_{\jmath}\int \mathrm{d}^3\boldsymbol{r}\int_0^{\infty}\mathrm{d}\tilde{\omega}\hbar\tilde{\omega}\hat{\boldsymbol{C}}_{\jmath}^{\dagger}(\boldsymbol{r}',\tilde{\omega})\cdot\hat{\boldsymbol{C}}_{\jmath}(\boldsymbol{r}',\tilde{\omega}). \tag{S23}$$

That is Eq. (5) of the main text. Now we use this Hamiltonian to obtain the dynamics of the excited atomic states.

9.2 Dynamical evolution of the excited atomic state.

In this section, we employ Schrödinger approach to evaluate the dynamical evolution of the excited atomic state. To this aim, we consider Schrödinger equation

$$\frac{\partial \left|\Psi(t)\right\rangle}{\partial t} = -\frac{\mathrm{i}}{\hbar} H \left|\Psi(t)\right\rangle , \tag{S24}$$

introduce the time-dependent amplitudes $c_{3(1)}(t)$ to $|3\rangle$ ($|1\rangle$) transitions and assume the Ansatz $|\Psi(t)\rangle$ as

$$\begin{aligned} \left|\Psi(t)\right\rangle =& \sum_{l=1}^{N_\mathrm{a}} c_{3,l} \left|3_l, \phi\right\rangle \otimes_{j\neq l} \left|1_j\right\rangle \\ &+ \sum_{\jmath,m} \int \mathrm{d}^3\boldsymbol{r} \int_0^\infty \mathrm{d}\tilde{\omega} c_{1j}(\tilde{\omega}, \boldsymbol{r}') \left|g, \boldsymbol{I}_{\jmath,m}\right\rangle , \end{aligned} \tag{S25}$$

where we assume $|\phi\rangle = |g_1, g_2, \ldots, g_N\rangle$ as a ground state of the atomic medium and

$$\left|\boldsymbol{I}_{\jmath,m}\right\rangle = \hat{C}^\dagger_{\jmath m}(\boldsymbol{r}', \omega) \left|\phi\right\rangle , \tag{S26}$$

as the excited plasmonic mode within the interface, respectively. Substituting Eqs. (S23) and (S25) into Eq. (S24), mapping

$$\frac{\mu_0\omega^2}{\pi} \boldsymbol{d}_\mathrm{a} \cdot \mathcal{A} \cdot \boldsymbol{d}_\mathrm{b} \sim g_\mathrm{ab}, \quad \sum_{\jmath m} \int \mathrm{d}^3\boldsymbol{r} \int_0^\infty \mathrm{d}\tilde{\omega} \mapsto \int_\mathrm{SPP}, \tag{S27}$$

the temporal evolution of the atomic transitions are

$$\frac{\partial c_1(t)}{\partial t} = \mathrm{i} \sum_{l=1}^{N_\mathrm{a}} c_3(t) g^*_\mathrm{ab}(\boldsymbol{r}_\mathrm{a}, \boldsymbol{r}'; \tilde{\omega}) \mathrm{e}^{\mathrm{i}(\tilde{\omega}-\omega_{31})t}, \tag{S28}$$

$$\frac{\partial c_3(t)}{\partial t} = \mathrm{i} \int_\mathrm{SPP} c_1(\boldsymbol{r}', \tilde{\omega}) g_\mathrm{ab}(\boldsymbol{r}_\mathrm{a}, \boldsymbol{r}'; \tilde{\omega}) \mathrm{e}^{-\mathrm{i}(\tilde{\omega}-\omega_{31})t}, \tag{S29}$$

Next, we perform direct integration of the (S28) and plug the resultant equation into (S29). Next, we employ

$$g_\mathrm{ab}(\boldsymbol{r}_\mathrm{a}, \boldsymbol{r}'; \tilde{\omega}) g^*_\mathrm{ab}(\boldsymbol{r}_\mathrm{a}, \boldsymbol{r}'; \tilde{\omega}) = \frac{\mu_0^2\tilde{\omega}^4}{\pi} (\boldsymbol{d}_\mathrm{a} \cdot \boldsymbol{\mathcal{A}} \cdot \boldsymbol{d}_\mathrm{b}) \times \left[\hbar\varepsilon_0 \mathrm{Im}[\varepsilon(\boldsymbol{r}', \tilde{\omega})] + \frac{\hbar^2\kappa_0^2}{\pi\tilde{\omega}^2} \boldsymbol{\nabla} \times \mathrm{Im}[\kappa(\boldsymbol{r}', \omega)]\right], \tag{S30}$$

for $g_{31} := g_\mathrm{ab} g^*_\mathrm{ab}$ and $\bar{\delta} = \tilde{\omega} - \omega_{31}$ to obtain the dynamics of the excited atomic states

$$\frac{\partial c_3(t)}{\partial t} = -\sum_l \int_0^\infty \mathrm{d}\tilde{\omega} \mathrm{Im}[g_{31}] \int_0^t \mathrm{d}\tau c_{3l}(\tau) \mathrm{e}^{\mathrm{i}\bar{\delta}(t-\tau)}. \tag{S31}$$

Consequently, performing the integration over the frequency deviation $\tilde{\omega}$ and substituting into Eq. (S31) then yields an integro-differential equation that describes the dynamics of the excited atomic states.

We evaluate the emitter-emitter strength coupling g_{31} in a wavenumber space. To this aim, we assume the atoms are doped to the interface in a height z_at. Consequently, the SPP wave can

couple to the atomic state only for $z \leq z_{\rm at}$. The generated spontaneous emission, then produces a SPP field with an arbitrary wavenumber $\boldsymbol{q}$ and a coupling function

$$g_{31} = \int \frac{\mathrm{d}^2\boldsymbol{q}}{(2\pi)^2} g_{\rm at}(\tilde{\omega};\boldsymbol{q})\mathrm{e}^{\mathrm{i}\boldsymbol{q}\cdot(\boldsymbol{r}_l-\boldsymbol{r}_j)}, \tag{S32}$$

similarly, we can represent the atomic-state amplitude in a wavenumber representation using a coupling function $\zeta^{(\rm C)}(\mathrm{C}(\boldsymbol{q},\boldsymbol{k}_{\rm SPP}) = \langle\Psi_{\boldsymbol{q}}|\Psi_{\boldsymbol{k}_{\rm SPP}}\rangle$.

Considering the dissipation of the SPP mode within the NIMM- quantum emitter interface as $\xi(\boldsymbol{q}) := \langle \boldsymbol{q}|\boldsymbol{L}\rangle := \mathrm{e}^{\mathrm{i}\boldsymbol{q}\cdot\boldsymbol{L}}$, the dynamics of the excited atomic state becomes

$$\frac{\partial c_3(t)}{\partial t} = -N_{\rm a}\int\frac{\mathrm{d}^2\boldsymbol{q}}{(2\pi)^2}\int \mathrm{d}\tilde{\omega}\mathrm{Im}[g_{\rm at}]\zeta^{(\rm C)}(\mathrm{C}(\boldsymbol{q},\boldsymbol{k}_{\rm SPP})\xi(\boldsymbol{q})\times\int_0^t \mathrm{d}\tau c_{3l}(\tau)\exp\{\mathrm{i}(\tilde{\omega}-\omega_{31})(t-\tau)\}. \tag{S33}$$

We assume the emitters are distributed within the interaction interface according to Gaussian distribution function

$$\zeta^{(\rm C)}(\boldsymbol{q},\boldsymbol{k}_{\rm SPP}) = \exp\left\{\frac{-L^2(\boldsymbol{q}-\boldsymbol{k}_{\rm SPP})^2}{2}\right\}. \tag{S34}$$

We also evaluate the green tensor related to the interaction interface using the Fourier dynamics characterized by complex frequency and real wavenumber for a perturbation frequency $\tilde{\omega} = \omega_{\rm SPP} - \mathrm{i}\gamma_{\rm SPP}$; $\gamma_{\rm SPP}$ the total relaxation of the system, by expanding the approach represented in[S8].

Consequently, we define the reduced green tensor $\mathcal{A}_{\rm SPP}(\boldsymbol{k}_{\rm SPP})$ as the residue of the $g_{\rm at}(\tilde{\omega},\boldsymbol{q})$ corresponds to pole $\tilde{\omega} = \omega_{\rm SPP}-\mathrm{i}\gamma_{\rm SPP}$ and use this tensor to describe the atomic evolution. This green tensor for our hybrid interface depends on the optical properties of the metamaterial layer $(\varepsilon_{\rm N},\mu_N)$, on propagation constant of dissipative $k_{\rm N}$ and atomic medium $k_{\rm a}$ $(k_{\imath'}^2 = k^2 - \tilde{\omega}^2\varepsilon_\jmath\mu_\jmath/\mathrm{c}^2)$, on unit vector of the SPP field $u_{\rm SPP}$ and is[S8]

$$\mathcal{A}_{\rm SPP} = \mathrm{i}\frac{k_{\rm a}\varepsilon_{\rm N}}{k_0\sqrt{\varepsilon_{\rm a}}}\frac{C(k,\tilde{\omega})}{2\mathrm{Re}[\omega_{\rm SPP}]}\times\left[\boldsymbol{e}_k - \frac{k}{k_{\rm N}}\boldsymbol{e}_z\right]\hat{\boldsymbol{u}}_{\rm SPP}(\boldsymbol{r}_l)\mathrm{e}^{\mathrm{i}(k_{\rm N}-k_{\rm a})z}. \tag{S35}$$

In our calculation we exploit the parallel component to achieve the emitter-emitter coupling and ignore the dispersive transverse component. Assuming the Lorentzian lineshape for spectral distribution, we evaluate $\mathrm{Im}[g_{\rm at}]$ in (S33) as

$$\mathrm{Im}[g_{\rm at}] = \frac{\mathcal{A}_{\rm SPP}^{\parallel}\gamma_{\rm SPP}}{(\tilde{\omega}-\omega_{\rm SPP})^2+\gamma_{\rm SPP}^2}. \tag{S36}$$

Finally, we substitute Eqs. (S34), (S36) and exploit $\mathrm{d}^2\boldsymbol{q} = q\mathrm{d}q\mathrm{d}\phi$ to perform integration over q. Then we achieve

$$-\frac{\partial c_3(t)}{\partial t} = \omega_{\rm o}^2\exp\{(t_{\rm L}\gamma_{\rm SPP})^2\}\int_0^t \mathrm{d}t'\ K(t,t')c_3(t'), \tag{S37}$$

with $t_L = L/v_{\rm SPP}$ is the SPP-flight time, $\tau_{\rm l} = \gamma_{\rm SPP}^{-1}$ is the time related to loss and $\omega_{\rm o}^2 := (N_{\rm a}/(2L)^2)g_{\rm at}$ and with

$$K(t,t') = \exp\left\{\frac{-(t-t'+2t_L^2\gamma_{\rm SPP})^2}{(2t_L)^2}\right\}. \tag{S38}$$

which is Eq. (7) in the main text. Note that in obtaining Eq. (S37), we employ two valid and widely used assumptions, namely, (i) we treat the Schrödinger approach to describe the dynamics

of the atomic ensemble, and (ii) we exploit the macroscopic Drude-Lorentz model to describe the optical frequency of our NIMM layer.

Using macroscopic model, we describe the electric permittivity ($\varepsilon_{\rm N}$) and magnetic permeability ($\mu_{\rm N}$) of the NIMM layer as

$$\varepsilon_{\rm N} = \varepsilon_\infty - \frac{\omega_{\rm e}^2}{\omega_l(\omega_l + {\rm i}\gamma_{\rm e})}, \tag{S39}$$

$$\mu_{\rm N} = \mu_\infty - \frac{\omega_{\rm m}^2}{\omega_l(\omega_l + {\rm i}\gamma_{\rm m})}, \tag{S40}$$

for ε_∞ and μ_∞ the background constant for the permittivity and permeability, respectively. The other constants are ω_l the perturbation frequency, $\omega_{\rm e}$ ($\omega_{\rm m}$) are the electric and magnetic plasma frequencies, and $\gamma_{\rm e}$ ($\gamma_{\rm m}$) are the corresponding decay rates. Specifically, we assume a nano-fishnet metamaterial layer fabricated with Al_2O_3-Ag-Al_2O_3 multilayer with rectangular nano-hole structure, which according to Ref.[S9] provides SPWs within optical frequency region. We exploit these parameters to describe the NIMM layer: $\varepsilon_\infty = \mu_\infty = 1.2$, $\omega_{\rm e} = 1.37 \times 10^{16}$ s^{-1}, $\omega_{\rm m} = 10^{15}$ s^{-1}, $\gamma_{\rm e} = 2.37 \times 10^{13}$ s^{-1} and $\gamma_{\rm m} = 10^{12}$ s^{-1}. This waveguide is low-loss for $|3\rangle \leftrightarrow |1\rangle$ (see main text for details of the realistic atomic medium and corresponding parameters) transition wavelength. Plugging into Eqs. (S35) and (S36), we can obtain the dynamics of the superradiant SPP dynamics within quantum emitter-NIMM layer interface when the signal and driving field is switched off.

10 Dynamical evolution of the weak SPP field in the presence of directional SSPP

In this section, first, we explore the dynamics of the SPP field within the interaction interface. This interface is coupled with a weak signal field, and a strong deriving field. The total plasmonic field related to the signal ($\boldsymbol{E}_{\rm s}$) and driving ($\boldsymbol{E}_{\rm d}$) lasers within the interaction interface is

$$\boldsymbol{E}(\boldsymbol{r}, t) = \sum_{m={\rm s,d}} \boldsymbol{E}_m(\boldsymbol{r}, t) + {\rm c.c.}, \tag{S41}$$

with

$$\boldsymbol{E}_m(\boldsymbol{r}, t) = \mathcal{E}_l \boldsymbol{u}_l(\boldsymbol{r}) \exp\{{\rm i}(\boldsymbol{k}\cdot\boldsymbol{r} - \omega_l t)\}, \tag{S42}$$

the electric field of the pumped lasers,

$$\boldsymbol{u}_m(\boldsymbol{r}) = c\left[k(\omega_l)\boldsymbol{e}_z - {\rm i}k_{\rm N}(\omega_l)\boldsymbol{e}_\parallel\right]/\varepsilon_0\omega_l, \tag{S43}$$

the unit signal ($\boldsymbol{u}_{\rm s}$) and driving ($\boldsymbol{u}_{\rm d}$) plasmonic field vectors along the interface. We define the amplitude of these fields as

$$\mathcal{E}_l = \left(\frac{\hbar\omega_l}{\varepsilon_0 L_x L_y L_z}\right), \tag{S44}$$

assume the interaction length along $x(y)$ direction as $L_x(L_y)$ direction and we define the field confinement factor as[S10]

$$L_z = \sum_{j={\rm N},0} \left\{ \left(\frac{\omega_l^2}{2c^2} \left[\frac{\tilde{\varepsilon}_j(|\boldsymbol{k}_j|^2 + |\boldsymbol{k}|^2)}{|\boldsymbol{k}_j||\varepsilon_j^2|} \right] + \frac{\tilde{\mu}}{2|\boldsymbol{k}_j|} \right) \right\}. \tag{S45}$$

Note that in defining Eq. (S45) we have defined

$$\tilde{\varepsilon}_j := {\rm Re}\left[\frac{\partial(\omega_l\varepsilon_j)}{\partial\omega_l}\right],\ \tilde{\mu}_j := {\rm Re}\left[\frac{\partial(\omega_l\mu_j)}{\partial\omega_l}\right], \tag{S46}$$

as effective electrical permeability and magnetic permeability of the interface, respectively.

The driving and signal fields are coupled to the atomic medium with Rabi frequencies $\Omega_{\rm d}$ and $\Omega_{\rm s}$ are tightly confined to the interface by transversely evanescence coupling functions $\zeta_{\rm d}(z)$ and $\zeta_{\rm s}(z)$ respectively (this coupling function is achieved in Refs.[S10,S11]. Specifically, we employ mapping

$$\Omega_m := \zeta_m(z)\Omega_m; m \in \{\rm d, s\}, \tag{S47}$$

and consider the effect of field confinement by exploiting field averaging

$$\langle \mathcal{F}(z)\rangle = \frac{\int_{-\infty}^{+\infty} {\rm d}z\, \zeta^*(z)\mathcal{F}(z)}{\int_{-\infty}^{+\infty} {\rm d}z\, |\mathcal{F}(z)|^2}. \tag{S48}$$

We assume the signal field to be weak and obtain its dynamics in the presence of directional superradiant emission. In what follows, we characterize the spatiotemporal dynamics of the signal plasmonic field within the interface and then we derive Eq. (13) of the main text.

10.1 Dynamics of weak plasmonic field: Maxwell-Schrödinger equation for our hybrid interface.

We achieve the dynamics of the weak SPP field, defined as $\Omega_{\rm s} := \boldsymbol{d}_{\rm l} \cdot \hat{\boldsymbol{E}}(\boldsymbol{r}, t)$ in a wavenumber representation as

$$\tilde{\Omega}_{\rm s} \approx \sum_{\jmath,m} \int \frac{{\rm d}^2\boldsymbol{q}}{(2\pi)^2} \int {\rm d}\tilde{\omega} \tilde{\mathcal{A}}_{13,m} C_{\jmath m}(\boldsymbol{q}, \omega){\rm e}^{{\rm i}(\boldsymbol{q}\cdot\boldsymbol{r} - \tilde{\omega}t)}. \tag{S49}$$

To perform the integration, first we should evaluate the dynamics of the quantized signal field $\hat{\boldsymbol{C}}_\jmath^{\rm s}$ in a wavenumber representation using the Heisenberg equation of motion. Also, this integration vanishes for the large perturbations in wave-vector and frequencies. Therefore for $\boldsymbol{q} \gg \boldsymbol{\mathcal{K}}$ ($\boldsymbol{q} \ll \boldsymbol{\mathcal{K}}$); $\mathcal{K}$ the dispersion of the signal plasmonic field, the signal SPP is highly dissipative due to atomic absorption. Consequently, we consider the resonant coupling between this plasmonic field and the atomic medium. To this aim, we choose

$$\boldsymbol{q} = \boldsymbol{k}_{\rm SPP} + \mathcal{O}(\boldsymbol{q} - \boldsymbol{k}_{\rm SPP}), \tag{S50}$$
$$\omega = \omega_{\rm SPP} + v_{\rm SPP}\delta\omega + \mathcal{O}(\delta\omega^2), \tag{S51}$$

and assume $\omega := \omega_{\rm SPP} + {\rm i}\gamma_{\rm SPP}$, $\mathcal{K} \approx k_{\rm SPP}$.

Now we can evaluate the plasmonic signal field in a Fourier space representation. Plugging Eqs. (S50) and (S51) into Eq. (S49), performing integration over $\tilde{\omega}$ by using $\delta\tilde{\omega} \ll \omega_{\rm SPP}$, defining $\tilde{\mathcal{A}}^{(\rm s)}_{13,m} = \mathcal{A}_{13,m}(\omega_{\rm SPP}, \mathcal{K})$ and $\delta\boldsymbol{q} = \boldsymbol{q} - \boldsymbol{\mathcal{K}}$, we achieve

$$\tilde{\Omega}_{\rm s} \approx \sum_{\jmath,m} \int \frac{{\rm d}^2\boldsymbol{q}}{(2\pi)^2} \tilde{\mathcal{A}}^{(\rm s)}_{13,m} C_{\jmath m}(\boldsymbol{q}, \omega){\rm e}^{{\rm i}(\delta\boldsymbol{q}\cdot\boldsymbol{r} - v_{\rm SPP}t)}. \tag{S52}$$

To achieve the spatiotemporal dynamics of (S52), first we investigate the dynamics of the $\hat{\boldsymbol{C}}_\jmath$ within quantum emitter-metamaterial interface. Considering the Hamiltonian of the system (S23), we achieve the equation of motion for $\hat{\boldsymbol{C}}_\jmath$ in terms of the atomic ground state $|G\rangle$ and excited state $|\Psi_0\rangle$ using the Heisenberg approach and defining

$$s(t) := \langle G| \sigma_l^- |\Psi_0\rangle + \langle \Psi_0| \sigma_l^+ |G\rangle \tag{S53}$$

as

$$\mathrm{i}\frac{\mathrm{d}C_{\jmath,m}}{\mathrm{d}t} = \tilde{\omega}C_{\jmath,m} - \sum_{l=1}^{N_\mathrm{a}} g^*_{\mathrm{at}}(\boldsymbol{r_l},\boldsymbol{r'};\tilde{\omega})\left[s(t)+s^*(t)\right], \tag{S54}$$

with $s(t)+s^*(t)\sim\rho_{31}(t)$. Here we assume the excited atom in this case is placed at $\boldsymbol{r}=\boldsymbol{r}_l$. Finally, we perform the derivative with respect to $\boldsymbol{r'}$ and time t from Eq. (S52) and make use of Eq. (S54) to achieve

$$\left(\frac{\partial}{\partial t}+\boldsymbol{v}_{\mathrm{SPP}}\cdot\boldsymbol{\nabla}\right)\Omega_\mathrm{s} = \mathrm{i}\mathcal{C}\tilde{\rho}_{31}, \tag{S55}$$

which is a Maxwell-Schrödinger equation for our plasmonic system. Eq. (S55) describe the evolution of the weak SPWs within a hybrid atomic medium-metamaterial interface.

10.2 Spatiotemporal evolution and mathematical derivation towards Maxwell-Schrödinger equation

In this section, we elucidate the spatiotemporal evolution for specific case of linearly polarized field and circularly polarized strong driving field. The Rabi frequency corresponds for the strong driving laser as a strong field with circular polarization

$$\Omega_\mathrm{d}(x,t) = \bar{\Omega}^{(1)}_\mathrm{d}(x,t)\hat{\epsilon}_+ + \bar{\Omega}^{(2)}_\mathrm{d}(x,t)\hat{\epsilon}_-, \tag{S56}$$

for right (ϵ_+), (ϵ_-) circular polarization and

$$\epsilon_\pm = (\boldsymbol{e}_x \pm \mathrm{i}\boldsymbol{e}_y)/\sqrt{2}. \tag{S57}$$

The Hamiltonian of the system in the presence of these two modulated fields is

$$\begin{aligned} H_\mathrm{I} =\hbar\Big[&\zeta^{(1)}_\mathrm{d}(z)\Omega^{(1)}_\mathrm{d}\mathrm{e}^{-\mathrm{i}\omega_{31}t}\left|3\right\rangle\left\langle 1\right| + \zeta^{(2)}_\mathrm{d}(z)\Omega^{(2)}_\mathrm{d}\mathrm{e}^{-\mathrm{i}\omega_{a1}t} \\ &\times\left|a\right\rangle\left\langle 1\right| + \zeta^{(1)}_\mathrm{d}(z)\Omega_\mathrm{s}\mathrm{e}^{-\mathrm{i}\omega_{31}t}\left|3\right\rangle\left\langle 1\right| + \mathrm{c.c.}\Big]. \end{aligned} \tag{S58}$$

for Ω_m the Rabi frequency of the signal and deriving fields

$$\Omega_\mathrm{s} = \frac{E_\mathrm{s}d_{31}}{\hbar}, \tag{S59}$$

$$\Omega^{(1)}_\mathrm{d} = \frac{E_\mathrm{d}d_{31}}{\hbar},\ \Omega^{(2)}_\mathrm{d} = \frac{E_\mathrm{d}d_{a1}}{\hbar}, \tag{S60}$$

we consider the relaxation rate of the $|1\rangle$ ($|3\rangle$) as γ_1 (γ_3), respectively, and assume the relaxation rate of the intermediate state as γ_a.

Next, we assume the following Ansatz for atomic transition amplitudes

$$\left|\psi(t)\right\rangle = c_1(t)\left|1\right\rangle + c_a(t)\left|a\right\rangle + c_3(t)\left|3\right\rangle. \tag{S61}$$

To achieve the dynamical evolution of the atomic states we assume simplification for field confinement

$$\zeta_\mathrm{s}(z)\approx\zeta_\mathrm{d}(z) := \zeta(z), \tag{S62}$$

employ mapping to deriving fields

$$\langle\zeta(z)\Omega_l\rangle \mapsto \Omega_l, \tag{S63}$$

and consider the rotated atomic transitions

$$c_3 \mathrm{e}^{-\mathrm{i}\omega_{31}t} \mapsto \tilde{c}_3,\ c_1 \mathrm{e}^{-\mathrm{i}\omega_{\mathrm{a},1}t} \mapsto \tilde{c}_1. \tag{S64}$$

Consequently, we evaluate the atomic state evolution as

$$\dot{c}_1(t) + \mathrm{i}\Omega_{\mathrm{d}}^{(1)} c_3(t) + \mathrm{i}\Omega_{\mathrm{d}}^{(2)} c_a(t) - \frac{\gamma_3}{2}c_3 - \frac{\gamma_\mathrm{a}}{2}c_a = 0, \tag{S65}$$

$$\dot{c}_3(t) + \left(\mathrm{i}\omega_{31} + \frac{\gamma_3}{2}\right) c_3(t) + \mathrm{i}\Omega_{\mathrm{d}}^{(1)} c_3 + \mathrm{i}\Omega_{\mathrm{d}}^{(2)} c_1 = 0. \tag{S66}$$

with

$$\tilde{\rho}_{31} = \left[c^*_{3,\mathrm{s}}(t)c_{1,\mathrm{d}}(t) + c^*_{3,\mathrm{d}}(t)c_{1,\mathrm{s}}(t)\right]\mathrm{e}^{-\mathrm{i}\theta-\gamma_{\mathrm{SPP}}t}, \tag{S67}$$

here $\varphi := \omega_{\mathrm{SPP}}t - \boldsymbol{\mathcal{K}}\cdot\boldsymbol{r}$. Finally we introduce signal ($\Delta_\mathrm{s} = \omega_\mathrm{s} - \omega_{31}$) and driving field detunings ($\Delta_\mathrm{d} = \omega_\mathrm{s} - \omega_{31}$) to achieve the temporal evolution of the atomic states. As it is evident from Fig. 1, atomic states $c_{\imath,\mathrm{s}}$; with $\imath \in \{1, \mathrm{a}, 3\}$ possess evolution due to both driving weak and signal fields illumination. In our analysis, we choose $\omega_{31} \approx \omega_{\mathrm{a}1}$. Consequently, the atomic transitions, coherence term and Rabi frequency of the driving fields are modified as

$$c_\imath = c_{\imath,\mathrm{s}} + c_{\imath,\mathrm{d}}, \tag{S68}$$

$$\tilde{\rho}_{31} = \tilde{\rho}_{31,\mathrm{s}} + \tilde{\rho}_{31,\mathrm{d}}, \tag{S69}$$

$$\Omega(\boldsymbol{r},t) = \Omega_\mathrm{s}(\boldsymbol{r},t) + \Omega_\mathrm{d}(\boldsymbol{r},t). \tag{S70}$$

The driving field is strong and hence we neglect its depletion due to amplification process. We also assume that the amplification for $\omega_{31} \approx \omega_{\mathrm{SPP}}$. Consequently, we consider the modulations due to weak signal field as

$$\Omega_\mathrm{s} = \zeta(z)\Omega_\mathrm{s}(t)\exp\{\mathrm{i}\varphi\}, \tag{S71}$$

$$c_{\imath,\mathrm{s}} = \zeta(z)c_{\imath,\mathrm{s}}(t)\exp\{\mathrm{i}\varphi\} + \mathrm{c.c.}, \tag{S72}$$

and also employ Fourier optics of SPP in our theoretical analysis. Now, we plug Eqs. (S68)-(S70) in Eqs. (S65) and (S66) and we use Eqs. (S71) and (S72) to characterize $\dot{c}_{\imath,\mathrm{s}}$, $c_\imath$. Next, we take derivative from Eq. (10) and replace $\dot{\tilde{\rho}}_{31}$ in terms of $\dot{c}_{\imath,\mathrm{s}}$, $c_\imath$. Finally, we achieve

$$\left(\frac{\partial}{\partial t} + \beta(x,t)\right)\left(\frac{\partial}{\partial t} + \boldsymbol{v}_{\mathrm{SPP}}\cdot\boldsymbol{\nabla}\right)\Omega_\mathrm{s} = \mathrm{i}\mathcal{C}f(x,t)\Omega_\mathrm{s}, \tag{S73}$$

here the coefficients β and f are

$$\beta := \tilde{\Delta} + \frac{\bar{\Omega}_\mathrm{d}^{(1)2} + \bar{\Omega}_\mathrm{d}^{(2)2}}{-\mathrm{i}\omega_{\mathrm{SPP}} + \gamma_{\mathrm{SPP}}} + \frac{4\bar{\Omega}_\mathrm{d}^{(1)}\bar{\Omega}_\mathrm{d}^{(2)*}\cos(2\varphi)}{\omega_{\mathrm{SPP}} + \mathrm{i}\gamma_{\mathrm{SPP}}},$$

$$f := 1 - \frac{2\bar{\Omega}_\mathrm{d}^{(1)2}}{\tilde{\Delta}(-\mathrm{i}\omega_{\mathrm{SPP}} + \gamma_{\mathrm{SPP}})} - \frac{2\bar{\Omega}_\mathrm{d}^{(1)2} + \bar{\Omega}_\mathrm{d}^{(2)2}\exp\{2\mathrm{i}\varphi\}}{\omega_{\mathrm{SPP}}(-\mathrm{i}\omega_{\mathrm{SPP}} + \gamma_{\mathrm{SPP}})}. \tag{S74}$$

that is Eq. (13) of our text.

Note that our quantitative description of the system and deriving Eq. (S73) is based on three assumptions: (i) employing the Schödinger equation to achieve atomic ensemble dynamics[S12], (ii) exploiting Drude-Lorentz model[S9,S13] to describe nano-fishnet metamaterial layer, and (iii) using Maxwell-Schrödinger equation[S14] to achieve the dynamics of the weak surface-plasmon polariton field. These are well-established methods to describe a nano-optic configuration and justify the validity of our theoretical model.

11 Calculation of the far-field superradiant field within interaction interface

In this section, we present the mathematical steps towards superradiant emission of radiation. We treat this superradiant plasmonic as far-field and employ the Dyadic green tensor to characterize this plasmonic field. We assume $X_{\mu\nu}$ to be this green tensor and characterize its components as

$$X_{xx}(\boldsymbol{k}_{\|}, z') := \mathrm{i}\frac{\mathcal{K}}{2k_2^3}\exp\{\mathrm{i}\mathcal{K}|z_{\mathrm{at}} - z|\}, \tag{S75}$$

$$X_{xz}(\boldsymbol{k}_{\|}, z') := \mathrm{i}\frac{k_{\|}}{2k_2^3}\exp\{\mathrm{i}\mathcal{K}|z_{\mathrm{at}} - z|\}, \tag{S76}$$

$$X_{yy}(\boldsymbol{k}_{\|}, z') := \mathrm{i}\frac{1}{2\mathcal{K}}\exp\{\mathrm{i}\mathcal{K}|z_{\mathrm{at}} - z|\}, \tag{S77}$$

$X_{xz} = X_{zx}$, $\tilde{g}_{\mu\nu} := g_{z_{\mathrm{at}},\mu\nu}$ and considering

$$\boldsymbol{k}_{\|} = k_x\boldsymbol{e}_x + k_y\boldsymbol{e}_y \tag{S78}$$

as

$$\tilde{g}_{xx} = \frac{1}{|\boldsymbol{k}_{\|}|^2}\exp\{-\mathrm{i}\mathcal{K}(z + z_{\mathrm{at}})\}\left[-k_x^2 r_{\mathrm{p}}^{k_x} X_{xx}(\boldsymbol{k}_{\|}, z') + k_y^2 r_{\mathrm{s}}^{k_y} X_{yy}(\boldsymbol{k}_{\|}, z')\right], \tag{S79}$$

$$\tilde{g}_{xy} = -\frac{k_x k_y}{|\boldsymbol{k}_{\|}|^2}\exp\{-\mathrm{i}\mathcal{K}(z - z_{\mathrm{at}})\}\left[r_{\mathrm{p}}^{k_x} X_{xx}(\boldsymbol{k}_{\|}, z') + r_{\mathrm{s}}^{k_y} X_{yy}(\boldsymbol{k}_{\|}, z')\right], \qquad \tilde{g}_{xy} = \tilde{g}_{yx}, \tag{S80}$$

$$\tilde{g}_{xy} = \frac{1}{|\boldsymbol{k}_{\|}|^2}\exp\{-\mathrm{i}\mathcal{K}(z + z_{\mathrm{at}})\}\left[-k_y^2 r_{\mathrm{p}}^{k_y} X_{xx}(\boldsymbol{k}_{\|}, z') + k_x^2 r_{\mathrm{s}}^{k_x} X_{yy}(\boldsymbol{k}_{\|}, z')\right], \tag{S81}$$

$$\tilde{g}_{xz} = -\frac{k_x}{|\boldsymbol{k}_{\|}|} r_{\mathrm{p}}^{k_x} X_{xz}(\boldsymbol{k}_{\|}, z')\exp\{-\mathrm{i}\mathcal{K}(z - z_{\mathrm{at}})\},\ \tilde{g}_{zx} = \frac{k_x}{|\boldsymbol{k}_{\|}|} r_{\mathrm{p}}^{k_x} X_{xz}(\boldsymbol{k}_{\|}, z')\exp\{-\mathrm{i}\mathcal{K}(z - z_{\mathrm{at}})\}, \tag{S82}$$

$$\tilde{g}_{yz} = -\frac{k_y}{|\boldsymbol{k}_{\|}|} r_{\mathrm{p}}^{k_y} X_{xz}(\boldsymbol{k}_{\|}, z')\exp\{-\mathrm{i}\mathcal{K}(z - z_{\mathrm{at}})\},\ \tilde{g}_{zx} = \frac{k_y}{|\boldsymbol{k}_{\|}|} r_{\mathrm{p}}^{k_y} X_{xz}(\boldsymbol{k}_{\|}, z')\exp\{-\mathrm{i}\mathcal{K}(z - z_{\mathrm{at}})\}, \tag{S83}$$

$$\tilde{g}_{yz} = \frac{|k_{\|}|}{\mathcal{K}} r_{\mathrm{p}}^{k_x} X_{xz}(\boldsymbol{k}_{\|}, z')\exp\{-\mathrm{i}\mathcal{K}(z - z_{\mathrm{at}})\}. \tag{S84}$$

Next, we define the polar coordinate in terms of azimuth (ϕ) and polar θ angles

$$\boldsymbol{e}_r := \sin\theta\left(\cos\phi\boldsymbol{e}_x + \sin\phi\boldsymbol{e}_y\right), \tag{S85}$$

we obtain the green tensor for a far-field superradiant SPP as

$$g_{\mu\nu}(\boldsymbol{r}, \boldsymbol{r'}, \mathcal{K}; \omega) = \mathrm{i}\frac{1}{2\pi}\left(\frac{\mathcal{K}z}{r^2}\right)\exp\left\{\mathrm{i}\boldsymbol{\mathcal{K}}\cdot(\boldsymbol{r} - \boldsymbol{r}')\right\} \times \tilde{g}_{\mu\nu}(\mathcal{K}\sin\theta\cos\phi, \mathcal{K}\sin\theta\sin\phi, \omega; z'). \tag{S86}$$

The electric field due to this far-field dyadic tensor is achieved by performing the integration over all possible times. Taking into account the relaxation rates of the atomic ensemble, this electric field relates to the phasing term

$$|\boldsymbol{S}| \sim |\tilde{g}_{\mu\nu}(\mathcal{K}\sin\theta\cos\phi, \mathcal{K}\sin\theta\sin\phi, \omega; z')|^2 \times \left|\sum_j^{N_{\mathrm{a}}}\exp\{\mathrm{i}\mathcal{K}\sin\theta(\cos\phi x + \sin\phi y) - \gamma_{\mathrm{R}} t\}\right|^2, \tag{S87}$$

indicates that the intensity pattern of the far-field superradiant SPP exist for the k_{SPP} satisfying the phase-match condition and also constructive interference of the superradiant field phase. These two conditions provides unique solution for the superradiant SPP intensity pattern that stands for *unidirectional* superradiant SPP generation.

References

S1. Strutt, M. J. O. Zur wellenmechanik des atomgitters. *Annel. Phys.* **391**, 319–324, DOI: https://doi.org/10.1002/andp.19283911006 (1928).

S2. Nayfeh, A. H. *Perturbation methods* (John Wiley & Sons, New York, 2008).

S3. Scully, M. O., Fry, E. S., Ooi, C. H. R. & Wödkiewicz, K. Directed spontaneous emission from an extended ensemble of n atoms: Timing is everything. *Phys. Rev. Lett. 96*, 010501, DOI: 10.1103/PhysRevLett.96.010501 (2006).

S4. Zhang, Y.-X., Zhang, Y. & Mølmer, K. Surface plasmon launching by polariton superradiance. *ACS Photonics 6*, 871–877 (2019).

S5. Philbin, T. G. Canonical quantization of macroscopic electromagnetism. *New J. Phys.* **12**, 123008, DOI: 10.1088/1367-2630/12/12/123008 (2010).

S6. Horsley, S. A. R. & Philbin, T. G. Canonical quantization of electromagnetism in spatially dispersive media. *New J. Phys.* **16**, 013030, DOI: 10.1088/1367-2630/16/1/013030 (2014).

S7. Marocico, C. A. & Knoester, J. Effect of surface-plasmon polaritons on spontaneous emission and intermolecular energy-transfer rates in multilayered geometries. *Phys. Rev. A* **84**, 053824, DOI: 10.1103/PhysRevA.84.053824 (2011).

S8. Archambault, A., Teperik, T. V., Marquier, F. & Greffet, J. J. Surface plasmon fourier optics. *Phys. Rev. B* **79**, 195414, DOI: 10.1103/PhysRevB.79.195414 (2009).

S9. Xiao, S., Chettiar, U. K., Kildishev, A. V., Drachev, V. P. & Shalaev, V. M. Yellow-light negative-index metamaterials. *Opt. Lett.* **34**, 3478–3480, DOI: 10.1364/OL.34.003478 (2009).

S10. Asgarnezhad-Zorgabad, S., Sadighi-Bonabi, R., Kibler, B., Özdemir, Ş. K. & Sanders, B. C. Surface-polaritonic phase singularities and multimode polaritonic frequency combs via dark rogue-wave excitation in hybridplasmonic waveguide. *New J. Phys.* **22**, 033008, DOI: 10.1088/1367-2630/ab7259 (2020).

S11. Asgarnezhad-Zorgabad, S., Sadighi-Bonabi, R. & Sanders, B. C. Excitation and propagation of surface polaritonicrogue waves and breathers. *Phys. Rev. A* **98**, 013825, DOI: 10.1103/PhysRevA.98.013825 (2018).

S12. Scully, M. O. & Zubairy, M. S. *Quantum optics* (Cambridge University Press, 1999).

S13. Kamli, A., Moiseev, S. A. & Sanders, B. C. Coherent control of low loss surface polaritons. *Phys. Rev. Lett.* **101**, 263601, DOI: 10.1103/PhysRevLett.101.263601 (2008).

S14. Svidzinsky, A. A., Yuan, L. & Scully, M. O. Quantum amplification by superradiant emission of radiation. *Phys.Rev. X* **3**, 041001, DOI: 10.1103/PhysRevX.3.041001 (2013).
